# Interpretable classifiers for prediction of disability trajectories using a nationwide longitudinal database

**DOI:** 10.1186/s12877-022-03295-x

**Published:** 2022-07-28

**Authors:** Yafei Wu, Chaoyi Xiang, Maoni Jia, Ya Fang

**Affiliations:** 1 The State Key Laboratory of Molecular Vaccine and Molecular Diagnostics, School of Public Health, Xiamen University, Xiamen, 361102 Fujian China; 2 National Institute for Data Science in Health and Medicine, Xiamen University, Xiamen, 361102 Fujian China; 3 Key Laboratory of Health Technology Assessment of Fujian Province, School of Public Health, Xiamen University, Xiamen, 361102 Fujian China; 4 School of Public Health, Xiamen University, Xiang’an Nan Road, Xiang’an District, Xiamen, 361102 Fujian China

**Keywords:** ADL limitations, Functional disability, Trajectories, Machine learning, Explanations

## Abstract

**Objectives:**

To explore the heterogeneous disability trajectories and construct explainable machine learning models for effective prediction of long-term disability trajectories and understanding the mechanisms of predictions among the elderly Chinese at community level.

**Methods:**

This study retrospectively collected data from the Chinese Longitudinal Healthy Longevity and Happy Family Study between 2002 and 2018. A total of 4149 subjects aged 65 + in 2002 with completed activities of daily living (ADL) information for at least three waves were included. The mixed growth model was used to identify disability trajectories, and five machine learning models were further established to predict disability trajectories using epidemiological variables. An explainable approach was deployed to understand the model’s decisions.

**Results:**

Three distinct disability trajectories, including normal class (77.3%), progressive class (15.5%), and high-onset class (7.2%), were identified for three-class prediction. The latter two were further merged into abnormal class, accompanied by normal class for two-class prediction. Machine learning, especially random forest and extreme gradient boosting achieved good performance in both two tasks. ADL, age, leisure activity, cognitive function, and blood pressure were key predictors.

**Conclusion:**

The findings suggest that machine learning showed good performance and maybe of additional value in analyzing quality indicators in predicting disability trajectories, thereby providing basis to personalize intervention measures.

**Supplementary Information:**

The online version contains supplementary material available at 10.1186/s12877-022-03295-x.

## Introduction

Disability has become a global problem characterized by restrictions on independent activities of daily living, which greatly reduces the individual's social adaptability and aggravates the economic burden [1–3]. The elderly are more likely to be accompanied by disability due to the decline of body function, the high incidence of diseases such as cerebrovascular diseases and rheumatoid arthritis, and the lack of necessary rehabilitation and self-care knowledge [4]. With rapid aging, the disabled population is also expanding. In China, the disability rate of people over 60 years old is about 26.4%, which is higher than that in the UK (18.3%) and in the US (15.6%) [5].

Disability level is constantly changing with age increases, especially for older adults [6]. In recent years, lots of studies [7–16] started to explore the distinct profile of disability trajectories in the elderly population and showed significant implications for developing personalized interventions for potential vulnerable older adults. For example, the pattern of disability trajectories is deeply explored among older adults in the United States [12, 14]. While trajectories of disability are yet to be fully investigated in older Chinese. With the development of health management and behavioral intervention, it is particularly important to identify the high-risk populations for developing unfavorable disability trajectories and take appropriate prevention measures in advance.

Machine learning (ML) is composed of a series of ingeniously conceived algorithms. It obtains features from large-scale data sets and automatically learns the internal patterns of data without any assumptions [17]. It is helpful to find complex nonlinear relationships in high-dimensional data. With the development of algorithms and the improvement of computing power, ML has been widely used in healthcare. Many studies have shown that ML methods can effectively improve the accuracy of disease diagnosis and prognosis prediction [17, 18]. Therefore, making full use of the advanced ML methods will help to improve the accuracy of disability trajectory prediction. However, most ML models are black-box models with defects in the visualization of intermediate processes and interpretation of results [19]. So its application in real-world clinical practice such as risk prediction models to assist medical decision-making is limited [20]. To overcome this problem, SHapley Additive exPlanations (SHAP) combined with the ML algorithms are performed for local and global interpretability analysis which provide intuitive explanations for risk prediction and are meaningful for early intervention [21].

In this context, this study focused on three issues: first, mixed growth model (GMM) was used to describe the heterogeneity of disability trajectories over a 16-year period among the 65 + adults of the Chinese Longitudinal Healthy Longevity and Happy Family Study. Second, under a consideration of feature selection (least absolute shrinkage and selection operator, LASSO), ML algorithms, namely logistic regression (LR), support vector machine (SVM), artificial neural network (ANN), and two ensemble learning methods of random forest (RF) and eXtreme Gradient Boosting (XGBoost), were used to predict the types of disability trajectories using epidemiological variables, and models were evaluated from three aspects of discrimination, calibration, and clinical usefulness. Third, the method of SHAP was applied to explain the potential mechanisms of the model’s decisions.

## Materials and methods

### Study design and participants

This study collected data from the Chinese Longitudinal Healthy Longevity and Happy Family Study (CLHLS-HF), which has been conducted by the Center for Healthy Aging and Development Studies, National School of Development of Peking University [22]. CLHLS-HF is the earliest and longest national social science survey in China. This project aims to investigate the determinants of health and life span of the elderly from various aspects. The survey covers 23 provinces, municipalities, and autonomous regions in China and the respondents are the elderly aged 65 and above and their children aged 35–64. A targeted random sample design is adopted to ensure the representativeness of the samples [23]. CLHLS-HF was approved by the Institutional Review Board, Duke University (Pro00062871), and the Biomedical Ethics Committee, Peking University (IRB00001052-13074). All participants provided written informed consent. All methods were carried out following the principles of the Declaration of Helsinki.

The six waves of data (2002, 2005, 2008–2009, 2011–2012, 2014, and 2017–2018) were used in this study. Participants aged 65 years and above in 2002 were selected at the baseline. In addition, in order to ensure that the follow-up information is sufficient to support the construction of trajectory model, we included respondents with at least three waves of completed ADL information, and finally 4149 subjects were included in this study. The follow-up information of CLHLS-HF database and the sample selection of this study are presented in Supplementary Fig. 1.

### Disability assessment

Disability was assessed by activities of daily living (ADL), which is consist of basic ADL (BADL) and instrumental ADL (IADL). BADL included the 6 daily activities (bathing, dressing, continence, using the toilet, indoor transferring, and feeding themselves), and IADL was measured by the 8 instrumental activities (shopping, cooking, visiting neighbors, doing laundry, walking continuously for 1 km, continuously crouching and standing up 3 times, lifting a weight of 5 kg, and taking public transportation). Each item was coded as 0 (“independently”), 1 (“with part assistance”), or 2 (“with complete assistance”). Consistent with previous studies [10, 13, 15, 16], disability scores were calculated by summing all 14 items so that a higher score for disability indicated worse physical condition. The disability scores were used to identify the potential trajectories of disability in the current analysis.

### Trajectories of disability

We used growth mixture model (GMM) to identify the heterogeneous disability trajectories of the targeted population from 2002 to 2018. GMM is able to establish several latent category groups considering individual and population heterogeneity, and the individuals in each category group enjoy the same or similar average growth trajectory (the same intercept and slope), which is used to describe the changes of individuals in category groups over time [24]. GMM estimates the parameters of the latent trajectories using the full-information maximum likelihood (FIML) approach, which allows incomplete longitudinal data [25]. Considering the sample size and the accuracy of characterization of disability trajectories, we selected the participants with at least three waves of disability data for analysis. Latent growth curve model (LGCM) and latent class growth model (LCGM) are special cases of GMM. LGCM assumes that all individuals come from homogeneous groups, which is often used to determine the shape of GMM (linear, quadratic, or cubic) [25]. When the latent category variable has only one level, GMM is simplified to LGCM. The optimal shape is determined by comparing the Bayesian information criteria (BIC) of LGCM (the smaller the better). LCGM considers group heterogeneity and divides the population into different trajectory categories, which can be further used to determine the number of potential categories of GMM [24, 26]. Referring to previous studies [27, 28], this study attempts to analyze the trajectories of 1–6 categories. The number of categories is selected according to some statistical indicators and practical significance. Statistical indices, including Bayesian information criteria (BIC), entropy, Vuong-Lo-Mendell-Rubin likelihood ratio test (VLMR-LRT), and proportion of the smallest class, were selected. BIC is an information criterion and its decline represents an improvement of the models. Entropy is a measure of classification accuracy, ranging from 0 to 1. The larger the entropy, the better the trajectories classification. VLMR-LRT compares the results of the k-1 class model with k class model. A significant *p-*value (< 0.05) indicates that k class model is better than k-1 class model. Besides, each class of trajectories must contain enough samples, no less than 5% of total population. In addition, the trajectory category should have practical significance, that is, easy to interpret and understand.

### Data collection and candidate variables

Predictors in this study included sociodemographic characteristics, lifestyles, objective examination, mental & cognitive & physical state, and family socioeconomic factors. For sociodemographic characteristics, age, sex (man, woman), ethnicity (Han ancestry, minority), education (illiterate, literate), occupation (low level, high level), marital status (in married, others [unmarried or separated or divorced or widowed]), residence (rural, urban), and co-residence (living alone, living with family) were included. Specifically, occupation was defined as a high level if the participant’s primary occupation before age 60 was professional, technical, governmental, institutional, managerial, or military personnel [29]. For lifestyles, fruits intake (low frequency, high frequency), vegetables intake (low frequency, high frequency), tea consumption (low frequency, high frequency), smoker (yes, no), alcohol drinker (yes, no), regular exercise (yes, no), and leisure activity index were considered. Especially, leisure activities included housework, personal outdoor activities, garden work, reading newspapers or books, raising domestic animals or pets, playing cards or mahjong, watching TV or listening to the radio, and taking part in some social activities. Each item had 5 levels and was coded as follows: “almost every day” (coded as 5), “not daily, but once a week” (coded as 4), “not weekly, but at least once a month” (coded as 3), “not monthly, but sometimes” (coded as 2), and “never” (coded as 1). A total score was calculated and then was divided by the full score to obtain a leisure activities index which is range from 0 to 1 [29]. Objective Examination included weight, systolic pressure, diastolic pressure, rhythm of heart (irregular, regular), length from wrist to shoulder, and length from kneel to floor. For mental state, psychological well-being (PWB) score were assessed, Mini-Mental State Examination (MMSE) score were selected for cognitive measurement, for physical state, BADL score, IADL score, chronic condition, and self-reported diagnosis of chronic diseases including hypertension (yes, no), diabetes (yes, no), heart disease (yes, no) and stroke (yes, no) were selected. PWB was measured by three positive items (self-reported life satisfaction, optimism, happiness) and three negative items (feel fearfulness or anxiety, feel lonely and isolated, feel useless with age). Specifically, for positive items, we coded as follows: 5 (“always”), 4 (“often”), 3 (“sometimes”), 2 (“seldom”), and 1 (“never”); and for negative items, we coded oppositely. Therefore, the total scores of PWB ranged 6–30, with a higher score indicating much better well-being [30]. Mini-Mental State Examination (MMSE) was used to assess global cognitive function and contains a total of 24 questions, involving 7 dimensions of orientation, food counting within one minute, memory, calculation, drawing, recall, and language. Except for food counting within one minute (one point for each food, and not exceed 7 points of a maximum score), other questions were coded as follows: 1 point (correct answer) and 0 point (wrong answer). So, the total scores of MMSE ranged from 0 to 30, with higher scores representing better cognitive function [27]. As for physical state, studies have also pointed out that the type and number of chronic diseases will affect the ability of daily living activities [31], so chronic condition and several diseases closely related to disability (hypertension, diabetes, heart disease, and stroke) were included. Chronic condition was measured by cumulative numbers of 11 chronic conditions, including hypertension, diabetes, cerebrovascular disease, heart disease, bronchitis/emphysema/asthma/pneumonia, tuberculosis, gastric/duodenal ulcer, cancer, arthritis, Parkinson's disease, and dementia [28]. For family socioeconomic factors, three predictors including household income per capita (low level, high level), adequate health services (yes, no), and sufficient financial support (yes, no) were selected. Specifically, the criteria for judging the level of household income per capita in rural and urban areas were 2,476 and 7,703 yuan respectively (approximately $299 and $931 based on the average exchange rate of RMB against US dollar in 2002) according to authoritative data released by the state. Those higher than this standard were defined as high level, while those below the criteria were defined as low level. Detailed measurements of variables were summarized in Supplementary Table 1. The multiple imputation approach (5 times) was applied to reduce the influence of missing values on predictors in the analysis.

### Feature selection

Feature selection helps to reduce the variable dimensions, thereby avoiding over-fitting of prediction models. In this study, least absolute shrinkage and selection operator (LASSO) was selected for feature selection. LASSO is a compression estimation method, which can perform variable selection and complexity regularization while fitting the generalized linear model [32]. LASSO models were constructed based on five sets of imputation data. The penalty coefficient of LASSO models was adjusted through threefold cross-validation, and then the features with parameter value (weight parameter) not equal to 0 were obtained in the predictive models.

### Machine learning classifiers

Logistic regression (LR) is a classical and commonly used risk prediction model. It explores the relationship between a group of independent variables and dependent variables with the use of Logit function and reflects the risk in the form of probability. It has the advantages of simple model setting, fast training speed, and good interpretation. Support vector machine (SVM) is a supervised learning method to realize the optimal division of data through the maximum interval hyperplane [33]. Because the result of SVM only depends on the support vector, it is especially suitable for the prediction of small sample data. SVM can map data to high-dimensional space with the help of kernel function to realize linear separation. In addition, the algorithm considers the minimization of empirical risk and structural risk and uses the hinge loss function as the agent loss, which leads to good stability [34]. Artificial neural network (ANN) refers to a complex network structure formed by a large number of neurons. It can approach nonlinear problem with strong self-learning and fault tolerance ability and can efficiently find the optimal solution for complex nonlinear problems [35]. The above three ML methods are all single classifiers, so their performance is limited in solving complex problems. Some studies have pointed out that combining multiple weak classifiers to achieve comprehensive prediction is helpful to improve the performance of the model, such as boosting and bagging [36, 37]. Boosting continuously adds the same weak learners in serial mode, adjusts the sample distribution according to the classifiers’ performance, and finally generates a strong learner in the form of voting or weighted average. Extreme gradient boosting (XGBoost) was selected as the representative in this study. Bagging realizes the prediction by synthesizing the results of multiple weak learners in a parallel way. Random forest (RF) method with decision tree as weak classifier was included. This method has the advantages of avoiding overfitting and automatic feature selection [38].

### Derivation and evaluation of prediction models

In this study, the data set was divided by nested cross-validation. This method can search for the best hyper-parameters and evaluate the performance of the model with cross-validation [39]. Specifically, two cycles, namely, outer loop and inner loop were performed in nested cross-validation. Inner loop aims to obtain the best hyper-parameters of the models. For this study, grid search was selected for searching hyper-parameters. Outer loop combined with optimal hyper-parameters was used to evaluate model’s performance subsequently. Through this strategy, the data leakage can be prevented to some extent, therefore obtaining a relatively low deviation of model evaluation. Some studies have pointed out that the test set error obtained by nested cross-validation is almost the real error [40]. In this study, ten-fold cross-validation was used in both inner and outer loops.

**Fig. 1:**
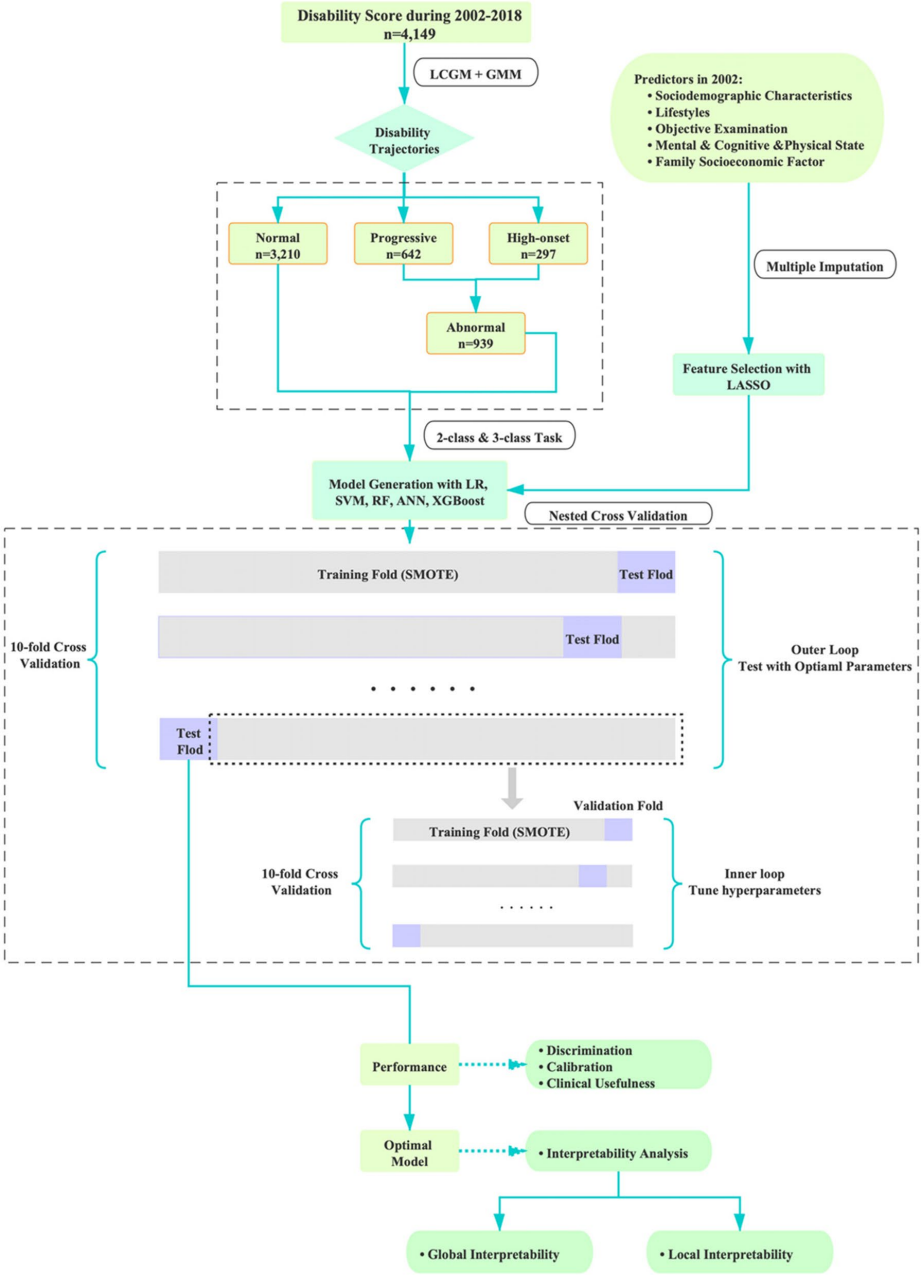
Flow chart of model derivation and validation

Considering the imbalance in outcome variable, synthetic minority over-sampling technique (SMOTE) [41] was used to increase the number of minority samples on the training set. For performance evaluation, discrimination, calibration, and clinical usefulness were assessed. Specifically, the indices for evaluating discrimination in this study included: (1) accuracy, which is used to evaluate the proportion of samples predicted correctly by the model to the total samples, (2) recall, which refers to the proportion of true positive samples predicted by the classifier to all positive samples, (3) precision, which is the correct proportion of the positive samples predicted by the classifier, (4) F1 score, which was the harmonic mean of precision and recall. In addition to these conventional ones, other comprehensive indices have been incorporated. Hamming distance measures the distance between the predicted label and the real label at the same position of the sequence. Jaccard similarity coefficient is an index to compare the similarity between limited sample sets. The value range of the above indicators is between 0 and 1, and a larger value indicates a better performance except for Hamming distance. Cohen's kappa score is an index to evaluate the consistency and reliability of classification results. Kappa takes the accuracy that would be generated purely by chance into account, which is essentially the ratio of actual consistency to non-opportunity consistency. It is less affected by unbalanced data with value ranging from -1 to 1 and the higher its value, the better the reliability of the result [42]. In addition, for the two-class prediction task, calibration was also assessed by reliability curve and histogram of prediction probability. The reliability curve is a curve with the predicted probability as the horizontal coordinate and the observed probability as the vertical coordinate. We hope that the predicted probability and the real value are as close as possible, and it is better that they are equal. Therefore, the closer the reliability curve and diagonal line of a model are, the better the calibration degree of the model is. Histogram of prediction probability is an image with the distribution of prediction probability after box division as the abscissa and the number of samples in each box as the ordinate. Also, decision curve analysis (DCA) was used to evaluate the clinical usefulness of prediction models in two-class task. DCA evaluates the net benefit of the model under different thresholds, which provides a basis for clinical decision-making. The line of “all positive” and “all negative” represent the extreme situation of net benefit when all subjects are all determined to be positive and negative. The flow chart of the model derivation and validation was shown in Fig. 1.

### Machine learning interpretation

SHAP was performed for interpretability analysis. This algorithm reflects the magnitude and direction of features’ influence on ML models by calculating the SHAP value, and makes local and global interpretability analyses for the targeted classification [21]. In local interpretability analysis, it shows the contribution of each feature to the prediction in a specific sample, in which the output is promoted from the base value (the mean of the predicted value) to the final value. Features that lower the SHAP value are shown in blue, otherwise in red. Global analysis includes summary plot and feature importance. The y-axis of summary plot indicates the feature ranking, and the x-axis represents the distribution of SHAP values that respond to features. In each row, the attribution of each individual to the outcome is drawn with dots of different colors, where red and blue color represent higher and lower feature values respectively. Feature importance chart is a bar chart obtained by taking the average of the absolute value of the feature's SHAP value (stacked bar charts for multi-classification).

### Sensitivity analysis

In this study, four sensitivity analyses were carried out to verify the stability of the results: (1) Given that trajectory analysis is more stable for participants with more observations over time, we conducted sensitivity analyses by restricting to participants with complete data for at least four waves (*n* = 2,457). We also compared the performance of ML models in distinguishing these trajectories. (2) Disability was measured by a composite score of 6 BADL and 8 IADL. However, the difficulty of completing each activity in IADL is more tough than in BADL, but the scores of each item are the same. Although the number of items in IADL is appropriately increased, it should be considered that IADL is more sensitive than BADL and more likely to reflect the changes of disability in earlier period [6, 43]. Therefore, we conducted trajectory analysis only based on the sum of 8 IADL score (*n* = 4,149), and prediction performance of this task was also compared with the original results. (3) Trajectories of disability may also be influenced by potential factors such as age, sex, and education [7–9, 31]. Therefore, with a consideration of age, sex, and education, we used these factors as covariates and explored the trajectories for the targeted participants (*n* = 4,149), and the ML models’ performance after considering covariates was also detected. (4) The missing rate of MMSE was about 40.49% in our analysis. We used multiple imputation to fill missing data and further included the participants with complete MMSE information for constructing prediction model (*n* = 2469) to investigate the impact of MMSE missing on the performance of the prediction models.

### Statistical analysis

Continuous variables were presented as mean ± standard deviation. Categorical variables were presented as percentages. The comparisons of baseline characteristics among different trajectories were performed by ANOVA test and chi-square test. All the above analyses were conducted with SPSS 27.0. Disability trajectories were performed with Mplus 8.3 (Muthén and Muthén, 2019). Feature selection, model derivation, and model evaluation were performed with Python 3.7.6. A two-sided *p*-value of < 0.05 was considered statistically significant.

## Results

### Heterogeneous trajectories of disability

A trajectory with quadratic shape yielded the best fit under LGCM because a lower BIC was observed. Among the LCGM models with classes ranging from 1 to 6, a three-class model was selected because the proportion of samples in the smallest group was less than 5% in four-class. Finally, a better fit (BIC = 102,109.346, entropy = 0.929, *p*-value of VLMR-LRT < 0.0001) and good separation of different classes (smallest class = 7.158%) were also observed in the three-class GMM model (Table 1).

**Table 1:** Performance of latent class growth model and growth mixture model

	BIC			
LGCM				
linear	105,530.680			
quadratic	105,345.372			
cubic	105,359.053			
LCGM	BIC	Entropy	VLMR-LRT	Smallest class
1-class	111,004.715			100%
2-class	105,054.609	0.942	0.0000	16.173%
3-class	102,817.976	0.892	0.0000	8.093%
4-class	101,585.221	0.904	0.0018	3.720%
5-class	100,586.689	0.881	0.0009	4.278%
6-class	99,933.216	0.862	0.0153	3.068%
GMM				
3-class	102,109.346	0.929	0.0000	7.158%

*Abbreviations*: *BIC* Bayesian information criteria, *VLMR-LRT* VUONG-LO-MENDELL-RUBIN likelihood ratio test, *LGCM* Latent growth curve model, *LCGM* Latent class growth model, *GMM*, growth mixture model

**Fig. 2:**
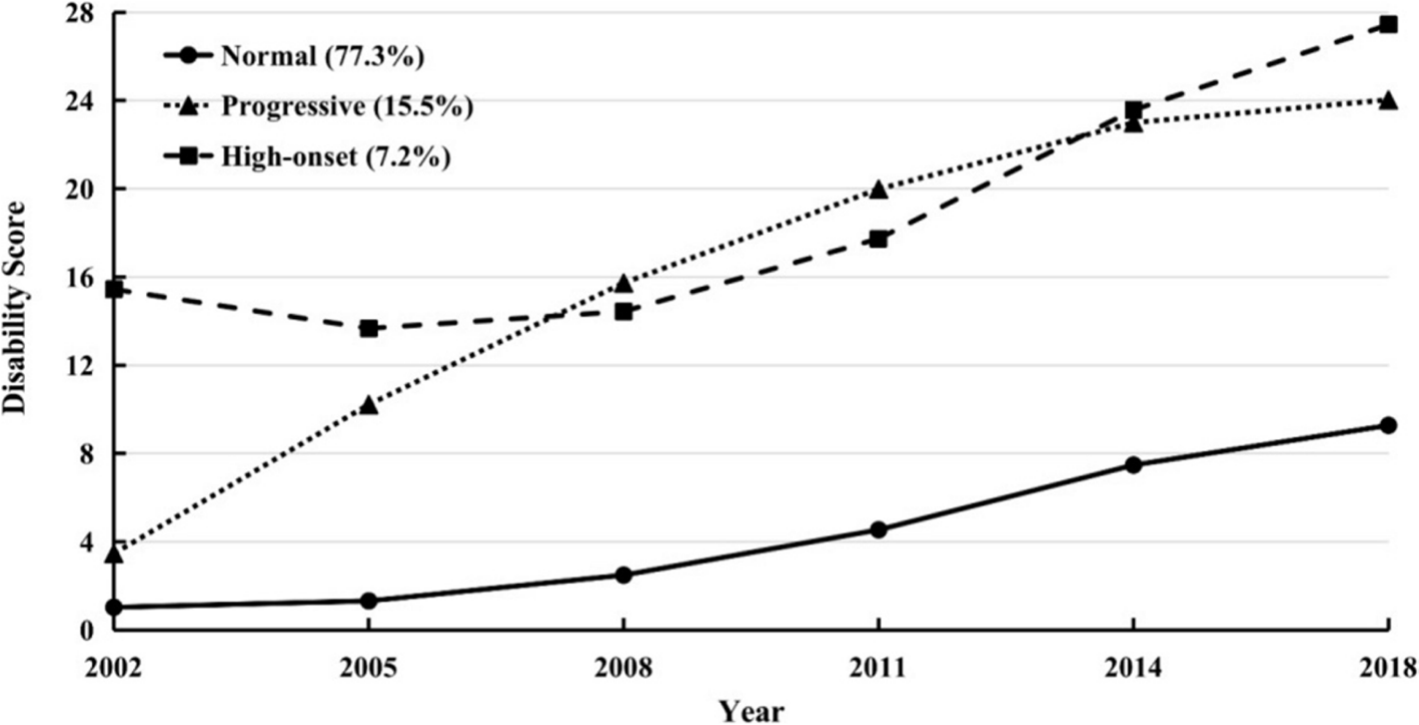
Heterogenous disability trajectory classes of older adults with complete information at least three waves (*n* = 4,149). Disability score (0–28) was measured by the sum score of BADL (0–12) and IADL (0–16). Three trajectory classes were identified: progressive class, high-onset class, and normal class. Each trajectory represents the mean change pattern of the 3 classes

Figure 2 shows the mean disability scores over time for the three trajectory classes: normal class (77.3%, intercept = 1.031, *p* < 0.001; linear slope = -0.152, *p* = 0.055; quadratic slope = 0.441, *p* < 0.001), progressive class (15.5%, intercept = 3.468, *p* < 0.001; linear slope = 7.383, *p* < 0.001; quadratic slope = -0.625, *p* < 0.001), and high-onset class (7.2%, intercept = 15.444, *p* < 0.001; linear slope = -3.035, *p* < 0.001; quadratic slope = 1.267, *p* < 0.001). The sensitivity analyses identified disability trajectories with similar shapes and distributions among participants who had complete information on ADL for at least four waves (Supplementary Fig. 2) and who had complete information on IADL for at least three waves (Supplementary Fig. 3). With adjusting age, sex, and education, similar results were also observed (Supplementary Fig. 4), and we found that there were differences in the baseline disability score between different genders (higher in woman compared with man, *p* < 0.001, Supplementary Table 2) as well as between different education level (higher in illiterate compared with literate, *p* < 0.001), but the effects of sex and education on the linear slope (*p* = 0.836, *p* = 0.622, respectively) and quadratic slope (*p* = 0.732, *p* = 0.243, respectively) were not significant. Meanwhile, age showed a positive effect on the disability score in each wave (all *p*-values < 0.001).

### Baseline characteristics of study population

Table 2 shows the results of baseline characteristics of the study samples with different trajectory classes. This study identified 3210, 642, and 297 participants with normal class, progressive class, and high-onset class, respectively. For the comparisons of baseline characteristics among trajectory classes, significant differences were found in all variables except for ethnicity, fruit intake, systolic pressure, length from wrist to shoulder, length from kneel to floor, PWB score, household income per capita, and sufficient financial support. Comparisons between the analytical sample and drop-out sample are presented in Supplementary Table 3. The samples in the current analysis were younger, more likely to live with family members and suffer from hypertension, had more females and rural residents, had lower frequency of fruit intake, had higher frequency of leisure activity and heart rate, had better basic activity of daily living, worse chronic condition, and a low level of household income.

**Table 2:** Sample characteristics by disability trajectories classes in 2002

	**Total sample (*N* = 4149)**	**Trajectory Class**			***P-*value**^a^	**Missing data**
		**Normal (*n* = 3210)**	**Progressive (*n* = 642)**	**High-onset (*n* = 297)**		
**Sociodemographic Characteristics**						
Age	74.99 ± 7.58	75.23 ± 7.97	85.42 ± 8.72	89.12 ± 9.30	< 0.001	—
Sex						
Male	1972 (46.4%)	1619 (50.4%)	248 (38.6%)	60 (20.2%)	< 0.001	—
Female	2222 (53.6%)	1591 (49.6%)	394 (61.4%)	237 (79.8%)		
Ethnicity						
Han Ancestry	3852 (92.8%)	2970 (92.5%)	602 (93.8%)	280(94.3%)	0.326	—
Minority	297 (7.2%)	240 (7.5%)	40 (6.2%)	17 (5.7%)		
Education						
Illiterate	2280 (55.1%)	1635 (51.0%)	412 (64.5%)	233 (79.3%)	< 0.001	10 (0.24%)
Literate	1859 (44.9%)	1571 (49.0%)	227 (35.3%)	61 (20.7%)		
Occupation						
Low level	497 (12.0%)	416 (13%)	63 (9.9%)	18 (6.1%)	0.003	12 (0.29%)
High level	3640 (88.0%)	2788 (87.0%)	574 (90.1%)	278 (93.9%)		
Marital Status						
Unmarried/Separated/Divorced/Widowed	2160 (52.1%)	1475 (46.0%)	451 (70.2%)	234 (78.8%)	< 0.001	—
Married	1989 (47.9%)	1735 (54.0%)	191 (29.8%)	63 (21.2%)		
Residence						
Urban	1692 (40.8%)	1242 (38.7%)	309 (48.1%)	141 (47.5%)	< 0.001	—
Rural	2457 (59.2%)	1968 (61.3%)	333 (51.9%)	156 (52.5%)		
Co-residence						
Living alone	664 (16.0%)	496 (15.5%)	135 (21.0%)	33 (11.1%)	< 0.001	—
With family	3485 (84.0%)	2714 (84.5%)	507 (79.0%)	264 (88.9%)		
**Lifestyles**						
Fruit Intake						
Low frequency	2758 (66.5%)	2124 (66.2%)	433 (67.4%)	201 (67.7%)	0.741	—
High frequency	1391 (33.5%)	1086 (33.8%)	209 (32.6%)	96 (32.3%)		
Vegetables Intake						
Low frequency	416 (10.0%)	285 (8.9%)	77 (12.0%)	54 (18.2%)	< 0.001	1 (0.02%)
High frequency	3732 (90.0%)	2924 (91.1%)	565 (88.0%)	243 (81.8%)		
Tea Consumption						
Low frequency	2797 (67.4%)	2097 (65.3%)	459 (71.5%)	241 (81.1%)	< 0.001	1 (0.02%)
High frequency	1351 (32.6%)	1112 (34.7%)	183 (28.5%)	56 (18.9%)		
Smoker						
Yes	979 (23.6%)	838 (26.1%)	117 (18.2%)	24 (8.1%)	< 0.001	1 (0.02%)
No	3169 (76.4%)	2371 (73.9%)	525 (81.8%)	273 (91.9%)		
Alcohol Drinker						
Yes	988 (23.8%)	822 (25.6%)	136 (21.2%)	30 (10.1%)	< 0.001	4 (0.10%)
No	3157 (76.2%)	2386 (74.4%)	505 (78.8%)	266 (89.9%)		
Regular Exercise						
Yes	1573 (37.9%)	1261(39.3%)	269 (42.0%)	43 (14.5%)	< 0.001	3 (0.07%)
No	2573 (62.1%)	1948 (60.7%)	372 (58.0%)	253 (85.5%)		
Leisure Activity Index	0.49 ± 0.13	0.51 ± 0.12	0.45 ± 0.13	0.31 ± 0.10	< 0.001	2 (0.05%)
**Objective Examination**						
Weight	51.25 ± 10.25	52.00 ± 10.02	49.65 ± 10.53	46.58 ± 10.43	< 0.001	—
Systolic Pressure	133.03 ± 17.63	132.35 ± 17.23	134.65 ± 18.15	136.80 ± 20.00	0.28	4 (0.10%)
Diastolic Pressure	84.65 ± 12.22	84.34 ± 12.06	85.79 ± 12.76	85.58 ± 12.57	0.04	6 (0.14%)
Rhythm of Heart						
Regular	3872 (93.4%)	3025 (94.3%)	588 (91.6%)	259 (87.5%)	< 0.001	4 (0.10%)
Irregular	273 (6.6%)	182 (5.7%)	54 (8.4%)	37 (12.5%)		
Heart Rate	72.44 ± 7.53	72.37 ± 7.46	72.73 ± 7.61	72.51 ± 8.10	0.01	6 (0.14%)
Length from Wrist to Shoulder	49.84 ± 5.53	50.20 ± 5.43	49.62 ± 5.90	48.33 ± 5.55	0.10	2 (0.05%)
Length from Kneel to Floor	46.66 ± 5.37	46.79 ± 5.27	46.49 ± 5.81	45.59 ± 5.28	0.14	6 (0.14%)
**Mental & Cognitive &Physical State**						
PWB Score	22.51 ± 3.74	22.64 ± 3.71	22.42 ± 3.57	20.97 ± 4.21	0.189	226 (5.45%)
BADL Score	11.77 ± 0.99	11.96 ± 0.26	11.81 ± 0.52	9.60 ± 2.70	< 0.001	—
IADL Score	13.80 ± 3.82	15.05 ± 1.85	12.60 ± 3.01	2.79 ± 2.44	< 0.001	—
MMSE score	27.95 ± 2.74	28.20 ± 2.40	27.08 ± 3.35	24.17 ± 5.12	< 0.001	1680 (40.49%)
Chronic Condition	0.68 ± 0.91	0.65 ± 0.85	0.77 ± 1.03	0.90 ± 1.09	< 0.001	364 (8.77%)
Hypertension						
Yes	677 (16.9%)	486 (15.7%)	126 (20.3%)	65 (22.3%)	0.001	145 (3.49%)
No	3327 (83.1%)	2606 (84.3%)	495 (79.7%)	226(77.7%)		
Diabetes						
Yes	77 (1.9%)	54 (1.7%)	19 (3.1%)	4 (1.4%)	0.07	146 (3.52%)
No	3926 (98.1%)	3044 (98.3%)	601 (96.9%)	281 (98.6%)		
Stroke						
Yes	178 (4.4%)	110 (3.5%)	36 (5.8%)	32 (11.1%)	< 0.001	125 (3.01%)
No	3946 (95.6%)	3005 (96.5%)	585 (94.2%)	256 (88.9%)		
Hear Disease						
Yes	363 (9.1%)	260 (8.4%)	71 (11.5%)	32 (11.1%)	0.02	141 (3.40%)
No	3645 (90.9%)	2841 (91.6%)	548 (88.5%)	256 (88.9%)		
**Family Socioeconomic factors**						
Household Income per Capita						
Low level	3029 (75.8%)	2337 (75.2%)	476 (77.7%)	216 (78.3%)	0.27	153 (3.69%)
High level	967 (24.2%)	770 (24.8%)	137 (22.3%)	60 (3.4%)		
Adequate Health Services						
Yes	3792 (91.4%)	2974 (92.7%)	586 (91.3%)	232 (78.1%)	< 0.001	1 (0.02%)
No	356 (8.6%)	235 (7.3%)	56 (8.7%)	65 (21.9%)		
Sufficient Financial Support						
Yes	3312 (79.9%)	2581 (80.4%)	508 (79.3%)	223 (75.1%)	0.08	2 (0.05%)
No	835 (20.1%)	628 (19.6%)	133 (20.7%)	74 (24.9%)		

Values are presented as mean ± standard deviation, number (%)

Abbreviations: PWB, psychological well-being; BADL, basic activity of daily living; IADL, instrumental activity of daily living; MMSE, Mini-Mental State Examination

^a^ANOVA test and chi-square test were performed, and the null hypothesis is no difference across the three classes

### Performance evaluation of three-class prediction models

The selected variables with LASSO in five sets of imputation data were shown in Supplementary Table 4, and the optimal hyper-parameters for each of the imputation data sets were listed in Supplementary Table 5. The performance of prediction models was shown in Table 3. Generally, the performance of five ML algorithms was comparable in the full-variable and selected-variable data sets. Specifically, XGBoost and RF achieved relatively the best performance, with balanced accuracy around 0.77, weighted recall around 0.84, weighted specificity and weighted F1 score nearly 0.85. Also, the two models performed moderately in other comprehensive indices with Hamming distance ranged 0.16–0.17, Jaccard similarity coefficient of 0.74 to 0.76, and Cohen’s kappa score in the 0.56–0.57 range. The SVM model was inferior to RF and XGBoost, while LR and ANN were the worst. The sensitivity analyses also showed similar results for three-class prediction among participants who had at least four waves of ADL information (Supplementary Table 6), and who had at least three waves of IADL information (Supplementary Table 7). Also, similar prediction performance was observed for predicting disability trajectories with consideration of covariates (Supplementary Table 8) as well as among participants who had complete information on MMSE (Supplementary Table 9).

**Table 3:** Performance of machine learning for three-class task prediction

	**Full variables**					**Selected variables with LASSO**				
	**LR**	**SVM**	**RF**	**ANN**	**XGBoost**	**LR**	**SVM**	**RF**	**ANN**	**XGBoost**
Accuracy	0.706	0.734	0.773	0.735	0.771	0.714	0.744	0.774	0.744	0.771
Recall	0.779	0.810	0.844	0.785	0.834	0.780	0.810	0.843	0.780	0.840
Precision	0.769	0.807	0.854	0.775	0.833	0.771	0.800	0.848	0.773	0.848
F1 Score	0.760	0.807	0.848	0.766	0.832	0.761	0.802	0.845	0.759	0.843
Hamming	0.221	0.190	0.156	0.215	0.166	0.220	0.190	0.157	0.220	0.160
Jaccard	0.639	0.705	0.759	0.645	0.735	0.639	0.696	0.754	0.636	0.751
Kappa	0.507	0.495	0.568	0.516	0.567	0.512	0.518	0.575	0.514	0.564

Accuracy, recall, precision, F1 score were all calculated with weighted metrics. Hamming, Jaccard, and Kappa refer to Hamming distance, Jaccard similarity coefficient, and Cohen’s kappa score

### Performance evaluation of two-class prediction models

**Fig. 3:**
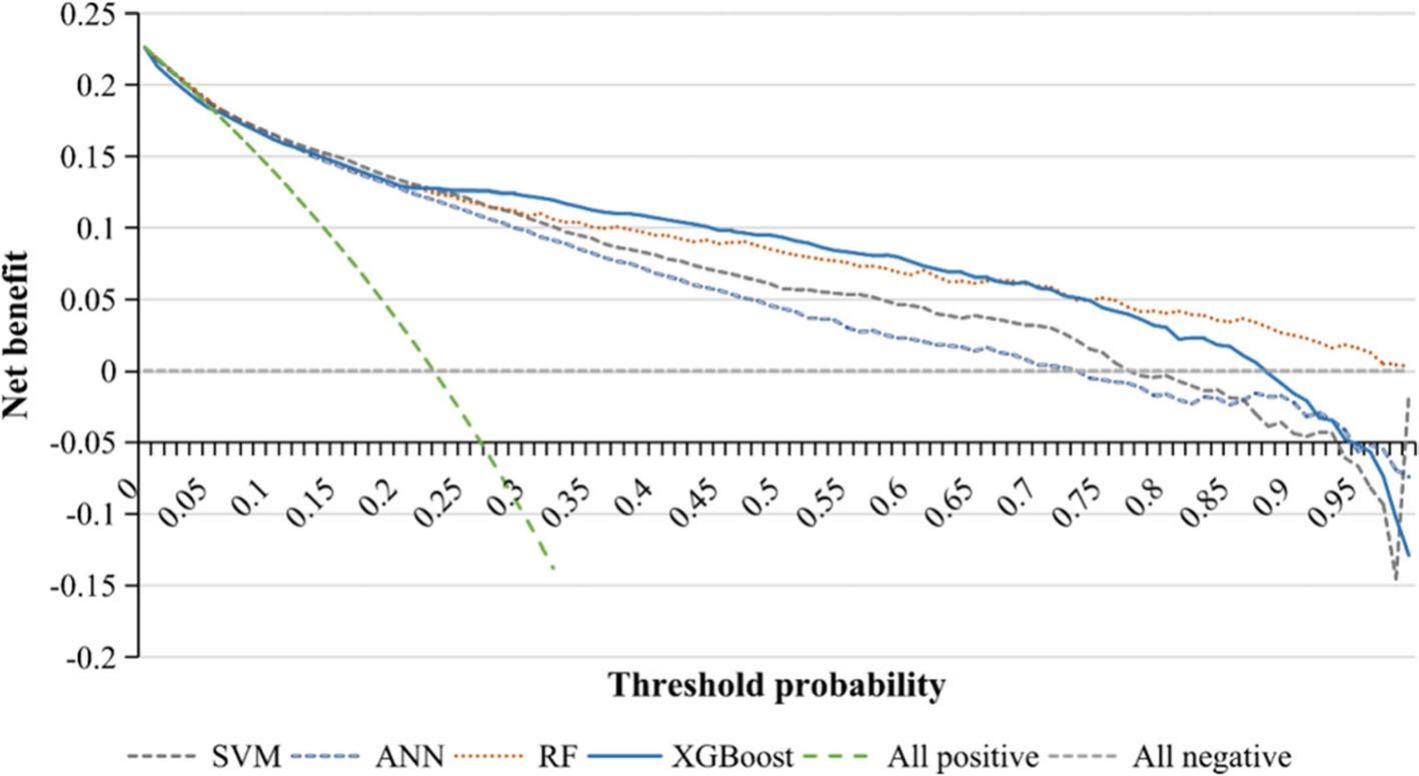
Decision curve analysis for the prediction models in full-variable data set

Considering the low proportion of progressive class and high-onset class, we further merged these two classes into abnormal class, so as to establish the two-class (abnormal class vs. normal class) ML models. Similarly, the original variables were processed by LASSO in the five sets of imputation data (Supplementary Table 10) and the hyper-parameters were tuned (Supplementary Table 11). Compared with the three-class task, the performance of ML models had a certain improvement in the binary classification task. Meanwhile, RF and XGBoost still had the best performance, followed by SVM, LR, and ANN (Table 4). Besides, the reliability curve and histogram of prediction probability demonstrated moderate calibration results (Supplementary Fig. 5–6). We observed that the reliability curves of RF and XGBoost were close to the diagonal line but the overall trend was lower than the line, which indicates that the predicted risk was slightly overestimated generally. The histogram showed that the prediction probability of most samples was concentrated near the value of 0, and there were only a few samples near the decision boundary (threshold probability at 0.5), indicating that the models had a high confidence degree. As presented in Fig. 3, the DCA curves of RF and XGBoost were far from the all-positive line and all-negative line and had higher net benefit within a large range of threshold probability compared with other prediction models, which suggested that RF and XGBoost had the best clinical value in predicting normal class vs. abnormal class. Similar results were also found in the data set with selected variables (Supplementary Fig. 7).

**Table 4:** Performance of machine learning for two-class task prediction

	**Full variables**					**Selected variables with LASSO**				
	**LR**	**SVM**	**RF**	**ANN**	**XGBoost**	**LR**	**SVM**	**RF**	**ANN**	**XGBoost**
Accuracy	0.760	0.776	0.805	0.748	0.810	0.764	0.770	0.784	0.753	0.796
Recall	0.828	0.843	0.859	0.816	0.861	0.831	0.837	0.847	0.821	0.854
Precision	0.824	0.841	0.867	0.813	0.870	0.828	0.833	0.850	0.818	0.860
F1 Score	0.821	0.841	0.862	0.808	0.864	0.823	0.832	0.848	0.813	0.855
Hamming	0.172	0.157	0.141	0.184	0.139	0.169	0.163	0.153	0.179	0.146
Jaccard	0.707	0.738	0.768	0.690	0.772	0.710	0.724	0.748	0.697	0.759
Kappa	0.554	0.564	0.583	0.532	0.586	0.564	0.565	0.560	0.542	0.573

Accuracy, recall, precision, F1 score were all calculated with weighted metrics. Hamming, Jaccard, and Kappa refer to Hamming distance, Jaccard similarity coefficient, and Cohen’s kappa score

### Interpretability analysis of prediction models

**Fig. 4:**
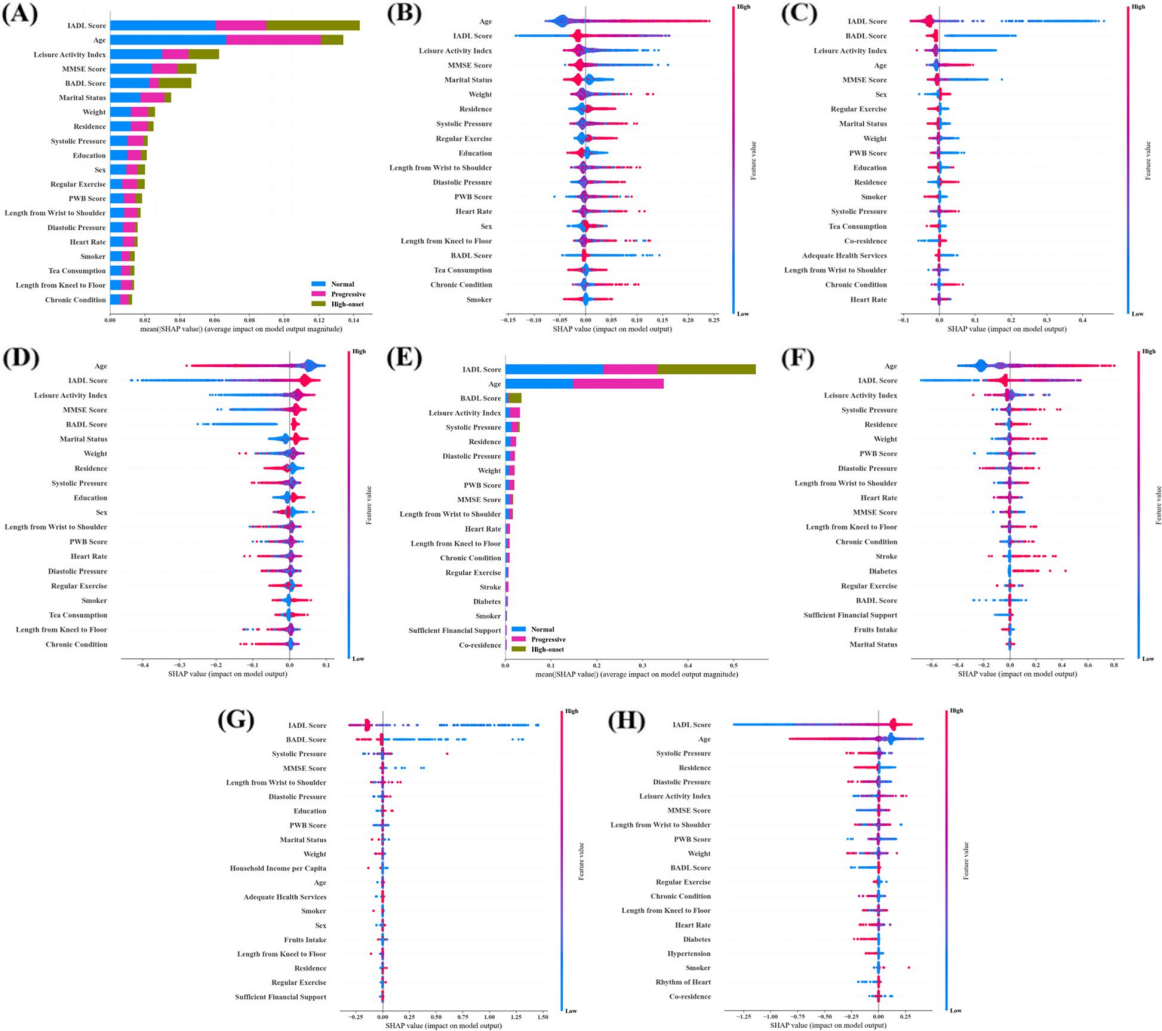
The relative feature importance (top 20) of RF (**A**-**D**) and XGBoost (**E**–**H**) in three-class prediction. **A**: overall importance of RF; **B** SHAP summary plot of RF model when the expected trajectory is progressive; **C** SHAP summary plot of RF model when the expected trajectory is high-onset; **D** SHAP summary plot of RF model when the expected trajectory is normal; **E** overall importance of XGBoost; **F** SHAP summary plot of XGBoost model when the expected trajectory is progressive; **G** SHAP summary plot of XGBoost model when the expected trajectory is high-onset; **H** SHAP summary plot of XGBoost model when the expected trajectory is normal

**Fig. 5:**
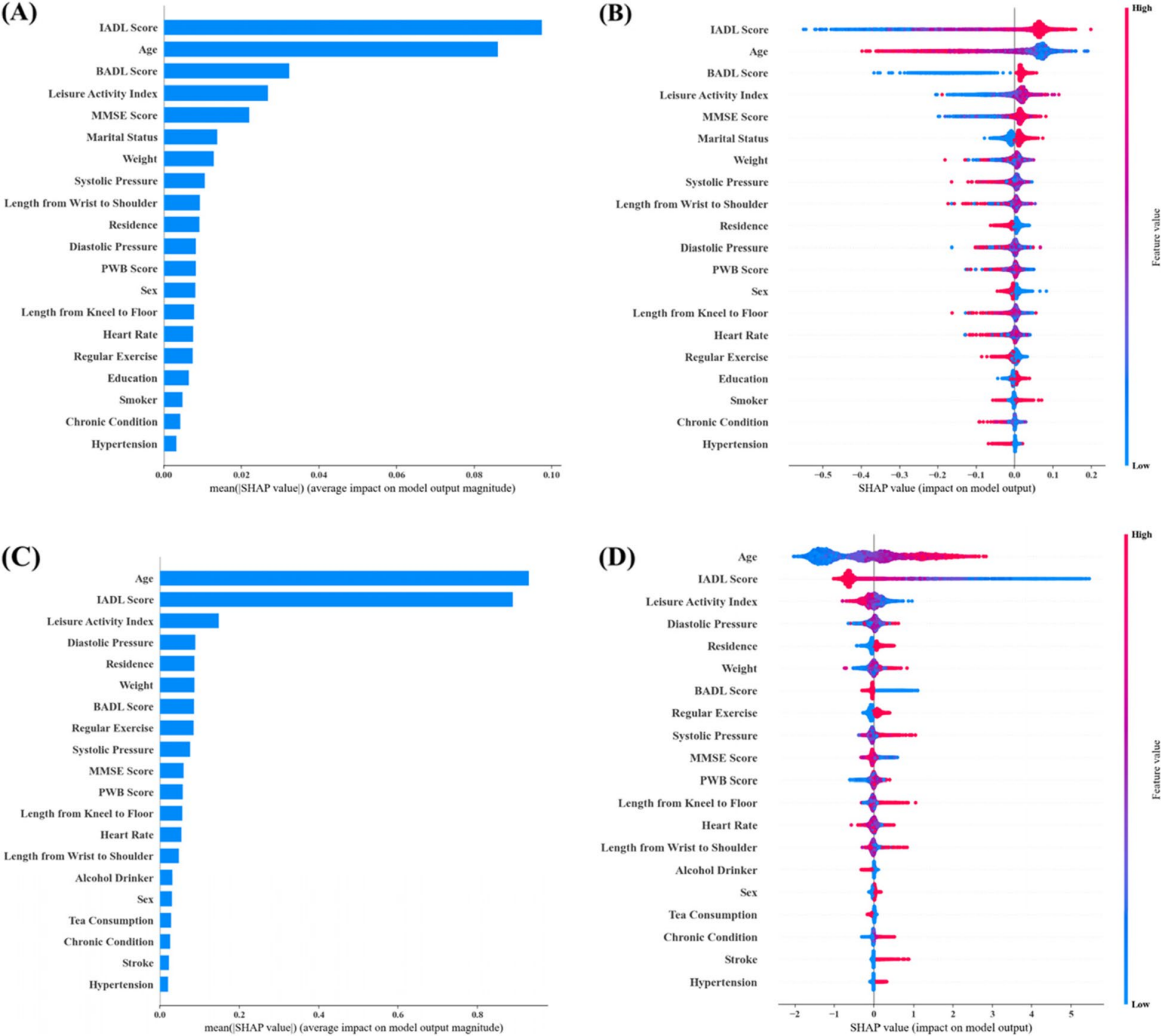
The relative feature importance (top 20) of RF (**A**-**B**) and XGBoost (**C**-**D**) in two-class prediction. **A** overall importance of RF; **B** SHAP summary plot of RF model when the expected outcome is abnormal; **C** overall importance of XGBoost; **D** SHAP summary plot of XGBoost model when the expected outcome is abnormal

The global interpretability method in SHAP was used to interpret the decisions for RF and XGBoost. For three-class task, the top 20 predictors for overall prediction and for prediction of each specific trajectory were shown in Fig. 4A-D (RF) and E–H (XGBoost). The top five most important predictors of RF were IADL, age, leisure activity, MMSE, and BADL. In XGBoost, IADL, age, BADL, leisure activity, and systolic pressure were the top five predictors. For two-class prediction, the top 20 predictors for overall prediction and for abnormal trajectory prediction were shown in Fig. 5 A-B (RF) and C-D (XGBoost). In terms of the top five factors in two-class prediction of RF, participants with older age, lower IADL score, BADL score, leisure activity index, and MMSE score were prone to experience an abnormal progress of disability. For the top five variables in XGBoost, older age, lower IADL score, leisure activity index, higher diastolic pressure, and living in urban areas were associated with a higher predicted probability of the abnormal class. Also, the predictors were similar in the LASSO-selected data set for three-class task (Supplementary Fig. 8A-D for RF; Supplementary Fig. 9A-D for XGBoost) and two-class task (Supplementary Fig. 10A-B for RF; Supplementary Fig. 11A-B for XGBoost).

**Fig. 6:**
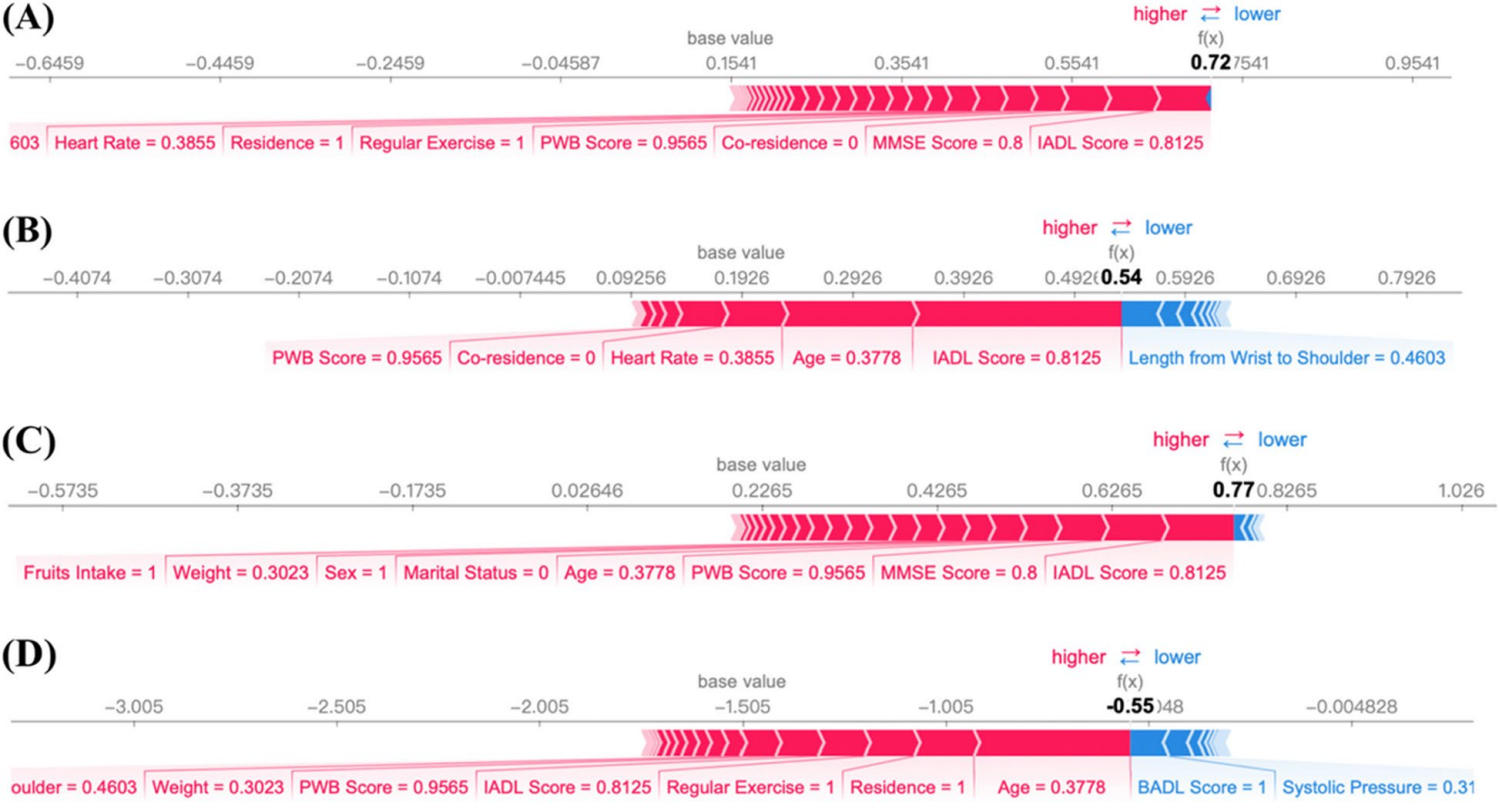
Local interpretation of samples based on RF and XGBoost. **A** RF explanation when the expected trajectory is progressive; **B** XGBoost explanation when the expected trajectory is progressive; **C** RF explanation when the expected trajectory is abnormal; **D** XGBoost explanation when the expected trajectory is abnormal

Furthermore, the local interpretability method in SHAP was used to understand the mechanism of individual prediction. For three-class prediction, we selected one sample with progressive disability trajectory to illustrate how RF and XGBoost work for making the final decision in full-variables data set. The output of SHAP value (0.72) in RF was higher than the base value (0.15, Fig. 6A), which was similar for XGBoost (Fig. 6B), therefore, the final predicted class was progressive trajectory. For two-class prediction, the results were shown in Fig. 6C-D when the selected sample was with abnormal class. Results for LASSO-selected data set were shown in Supplementary Fig. 12A-D.

## Discussion

Based on nationally representative samples of older Chinese community-dweller, this study extracted disability trajectories from 4149 subjects by GMM, which were divided into three categories: normal, progressive, and high-onset. The shape and trend of these three kinds of trajectories are similar to previous studies [3, 9, 12, 14]. Among them, the normal category represents the development trajectory of normal people, showing a slow upward trend, mainly caused by the decline of body function with the increase of age [31]. The progressive class shows a trend that starts with an emergency and then slows down. The emergence of this group is mostly attributed to some diseases or injuries that restrict physical activities, such as cerebrovascular diseases and rheumatoid arthritis [44]. Some studies have also pointed out that the type and number of chronic diseases also affect the ability to perform activities of daily living to varying degrees [31]. While in the later period, the progression of disability is slowing due to the intervention of treatment and rehabilitation. High-onset groups are in a relatively serious disability state at the baseline, showing a slow development trend before urgent. This group accounts for the least. The possible explanation for their existence is that this group can better manage the disability state in the early phase, but in the later stage, due to the accumulation of disability risk factors and the lack of awareness of long-term health management, a rapid development speed appears.

The progressive and high-onset classes are both unfavorable functional states. Considering the relatively small proportion of these two classes, we combined them into abnormal class. Although studies have proved that binary prediction reduces the amount of outcome information, ambiguous prediction could provide greater confidence and improve the prediction performance to certain extent [45]. In this study, five ML methods were included to carry out three-class and two-class outcome predictions on the whole data set and the feature selection data set screened by LASSO. We found that the performance of the two-class prediction was better than that of the three-class prediction in both data sets, which confirms the above conclusion. Furthermore, it could be found that although LASSO compressed the number of variables, there was no significantly decline of the models’ performance, suggesting that fewer key variables selected by LASSO was also able to achieve the prediction effect similar to the full-variable data set. In addition, we discovered that the ensemble learning method (RF and XGBoost) had the best performance among the five models, which is attributed to the fact that it form a stronger classifier by combining multiple weak learners [37], even if some weak ones get wrong predictions, the others can also correct the error to varying degrees [36].

Most of ML algorithms are known to be black-box models, which restricts the application of the ML methods in medical decision-making [20]. To overcome this problem, we used SHAP to analyze the local and global interpretability of the models’ decisions, aiming to provide references for the practical application of the models. In general, baseline ADL, age, leisure activity, cognitive function, and blood pressure showed significant influence on the prediction of disability trajectories. Disability level is constantly progressing as age increases [4, 11]. Some researchers have shown that leisure activity is strongly associated with the risk of disability [46, 47]. Cognitive impairment has a significant impact on disability and they are mutually reinforcing over time [48, 49]. In addition, hypertension can increase the risk of motor, cognitive, and mood disorders, and thus affect patients' ability to perform activities of daily living [50]. Baseline ADL score is not only a component of the disability trajectories, but also an important predictor in following trajectory prediction. The use of baseline ADL score for prediction is consistent with real-world applications because the trajectories were also composed by the data of other five waves and ADL information available at baseline can be used to predict some future outcome. Similar idea was also validated in several successful cases [51–53], in which the influential predictor was important constitution of outcome on the machine learning prediction. It is worth noting that baseline IADL and BADL are the two most important predictors when the expected outcome is high-onset, which is largely related to the stratification of intercept between high-onset and other two types of trajectories, and patients with severe disability state at baseline are more likely to continue to suffer from worse functional condition. The importance of baseline IADL and BADL decrease in distinguishing normal and progressive class for their similar baseline scores, thus, other variables are needed to make more accurate predictions, such as leisure activity and age. Surely, the interpretability analyses were performed to provide internal explanations for models’ predictions, rather than exploring causality between predictive variables and outcome because the goal of prediction is distinct from causal inference, it mainly focuses on making effective prediction of disability trajectories using advanced machine learning methods, and it maybe of additional value in discovering potential risk factors [54].

The study focused on the dynamic process of disability, rather than the static disability condition. The two-class and three-class prediction models are applicable to different scenarios. If more attention is paid to the long-term outcomes in clinical practice, the two-class model with better performance will be the first choice. If more emphasis is placed on early prevention and early intervention, the three-class model with more detail information can provide more specific guiding significance. It should be noted that the abnormal class of the binary models includes trajectories of progressive and high-onset, and the baseline ADL of these two categories is significantly different, but the baseline ADL cannot be used as the criterion for the classification of these two categories, because the trajectory models only provide an average description [24, 25]. Due to individual variation, the starting point of the trajectory is not fixed at individual level, so the identification of these two trajectories still needs to be judged according to the output of the prediction model.

The practical significance of this study is to provide more accurate prediction tools for primary screening of high-risk populations at a community level. We emphasize the use of easily available, low-cost epidemiological variables as predictors. Although some studies have pointed out that the inclusion of longitudinal time series data can improve the performance of prediction models [55, 56], we are more concerned about the convenience and accessibility of data collection, which is more in line with the requirements of practice. In addition, the identified important variables provide guidance for prevention strategies of the vulnerable population, such as regular recreational activities, cognitive training, and blood pressure management. This result also suggests that individuals with poor ADL and older age are the key groups for prevention of disability, and effective management and support of these high-risk groups is necessary to achieve better gains of population health.

## Limitations

There are still some shortcomings in the current study. Firstly, ML methods are superior in dealing with high-dimensional and nonlinear problems, which depend on the support of sufficient sample size [17]. However, the sample size included in this study was relatively small, which might limit the performance of the models. Given this condition, we used nested cross-validation to make full use of available data and selected various metrics for performance evaluation. Secondly, the missing rate of MMSE was relatively high, which might have impact on predictions of ML models. To minimize the impact, multiple imputation and sensitivity analysis were implemented. Thirdly, the current study only carried out internal validation, lacking external validation to evaluate the models’ generalization ability, so we will further expand the scope of the study to include samples from different regions for external validation. Finally, the prediction models in our study have not been transformed into clinical application. We discussed the application prospect of the models in clinical practice, and subdivided the models for two-class and three-class prediction according to different application scenarios, providing sufficient preparation for clinical application. Furthermore, appropriate tools such as risk calculators should be developed for achieving social and economic benefits.

## Conclusion

Three distinct disability trajectories were found in Chinese older community-dwellers. ML methods, especially RF and XGBoost, had good performance in distinguishing both two-class and three-class tasks. Meanwhile, the interpretability analysis based on SHAP found that baseline ADL score, age, leisure activity, cognitive function, and blood pressure were the most important predictors. In clinical application, the two-class and three-class predictions are suitable for different scenarios, and under the guidance of important predictors, personalized intervention measures, such as regular recreational activities, cognitive training, and blood pressure management, can be formulated towards people at high risk of disability.

## Supplementary Information

**Additional file 1.**

## Data Availability

Data supported for the current study was publicly available at the Chinese Longitudinal Healthy Longevity and Happy Family Study (https://opendata.pku.edu.cn/dataverse/CHADS).

## Abbreviations

ADL: Activities of daily living
ML: Machine learning
SHAP: SHapley Additive exPlanations
GMM: Mixed growth model
CLHLS-HF: Chinese Longitudinal Healthy Longevity and Happy Family Survey
LR: Logistic regression
SVM: Support vector machine
ANN: Artificial neural network
RF: Random forest
XGBoost: Extreme gradient boosting
SMOTE: Synthetic minority over-sampling technique
DCA: Decision curve analysis
