# Correction: Interpretable classifiers for prediction of disability trajectories using a nationwide longitudinal database

**DOI:** 10.1186/s12877-023-03843-z

**Published:** 2023-03-30

**Authors:** Yafei Wu, Chaoyi Xiang, Maoni Jia, Ya Fang

**Affiliations:** 1 The State Key Laboratory of Molecular Vaccine and Molecular Diagnostics, School of Public Health, Xiamen University, Xiamen, 361102 Fujian China; 2 National Institute for Data Science in Health and Medicine, Xiamen University, Xiamen, 361102 Fujian China; 3 Key Laboratory of Health Technology Assessment of Fujian Province, School of Public Health, Xiamen University, Xiamen, 361102 Fujian China; 4 School of Public Health, Xiamen University, Xiang’an Nan Road, Xiang’an District, Xiamen, 361102 Fujian China

**Correction: BMC Geriatr 22, 627 (2022)**

**https://doi.org/10.1186/s12877-022-03295-x**

After publication of this article [1], the authors reported that in the Results section, in Table 2, and in the Additional file 1 several corrections should be made. These are summarized in the accompanying Supplementary file.

The original article [1] has been corrected.

## Supplementary Information

**Additional file 1.** Revised content in main text.
