# Supplementary material for: Interpretable classifiers for prediction of disability trajectories using a nationwide longitudinal database
Source: BMC Geriatr. 2022 Jul 28;22:627. doi: 10.1186/s12877-022-03295-x (PMC9336105; doi:10.1186/s12877-022-03295-x)
Supplement: Supplementary file 1 — Additional file 1. [file 12877_2022_3295_MOESM1_ESM.docx]

**Interpretable classifiers for prediction of disability trajectories using a nationwide longitudinal database**

**Wave 2002 (n=16,064)**

**Wave 2005 (n=15,638)**

**Wave 2008 (n=16,540)**

**Wave 2011(n=9,765)**

**Wave 2014 (n=7,192)**

**Wave 2018 (n=15,874)**

**7,889 lost to follow up**

**7,463 newly recruited**

**8,166 lost to follow up**

**9,068 newly recruited**

**8,307 lost to follow up**

**1,532 newly recruited**

**3,698 lost to follow up**

**1,125 newly recruited**

**3,729 lost to follow up**

**12,411 newly recruited**

**n=16,064**

**n=4,146**

**Selected 2002 as baseline**

**11,915 excluded according to following criteria:**

- **aged< 65 years;**
- **Completed < 3 waves of ADL between 2000-2018.**

**Supplementary Fig. 1 |** Flow chart of CLHLS-HF survey and sample selection in current analysis.


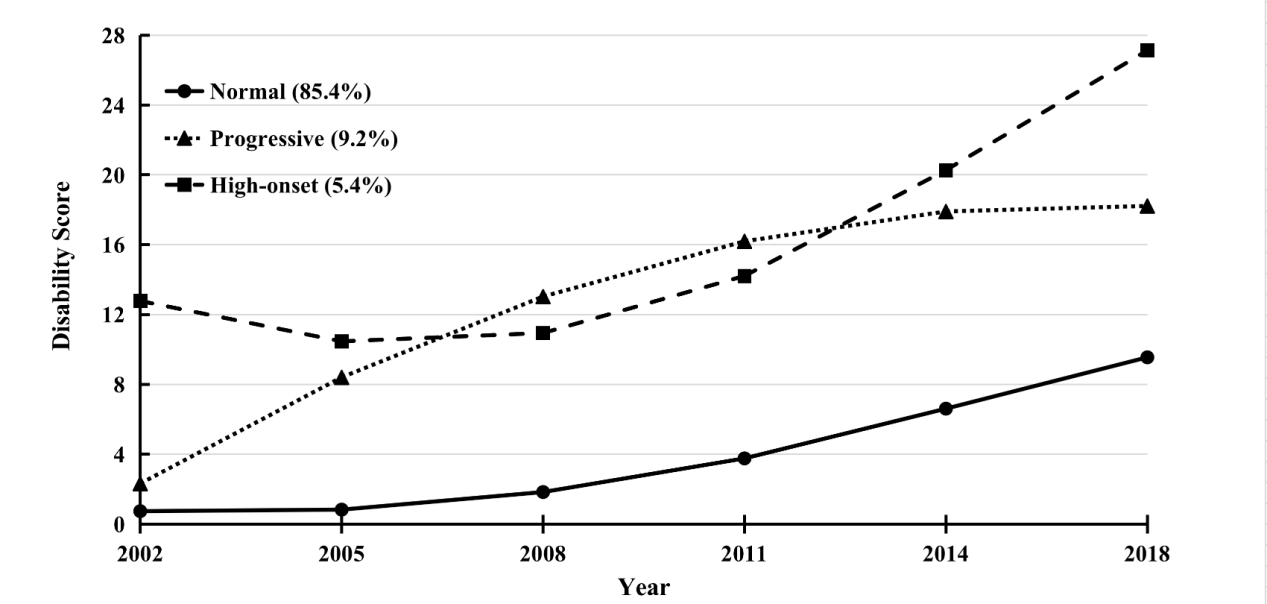


**Supplementary Fig. 2 |** Heterogenous disability trajectory classes of older adults with complete information for at least four waves (n= 2,457). Disability score (0-28) was measured by the sum score of BADL (0-12) and IADL (0-16). The three-class GMM was selected as final model (Bayesian information criteria= 68,354.452; entropy= 0.959; Vuong-Lo-Mendell-Rubin Likelihood Ratio Test p=.003), including “Normal” class (intercept= 0.743, *p*<0.001; linear slope= -0.373, *p*<0.001; quadratic slope= 0.460, *p*<0.001), “Progressive” class (intercept= 2.325, *p*<0.001; linear slope= 6.814, *p*<0.001; quadratic slope= -0.730, *p*<0.001), and “High-onset” class (intercept= 12.780, *p*<0.001; linear slope= -3.707, *p*<0.001; quadratic slope= 1.395, *p*<0.001). It showed similar pattern of disability trajectory classes with our analytic sample (n= 4,149).

**
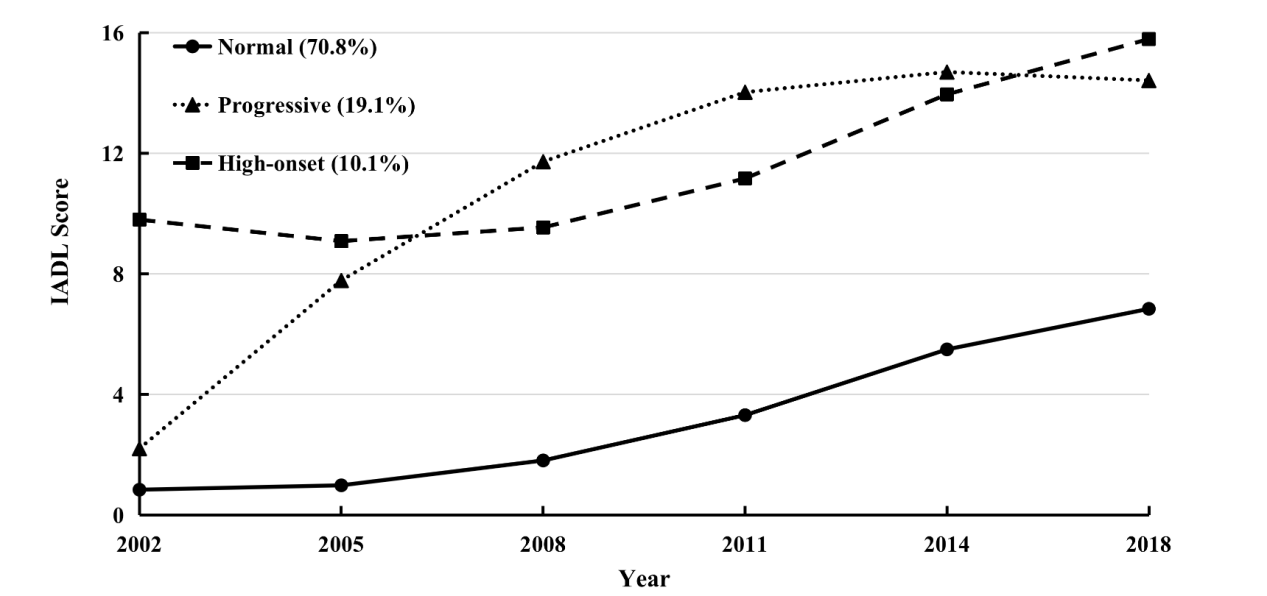
**

**Supplementary Fig. 3 |** Heterogenous IADL (0-16) trajectory classes of older adults with complete information for at least three waves (n= 4,149). The three-class GMM was selected as final model (Bayesian information criteria= 94,589.351; entropy= 0.927; Vuong-Lo-Mendell-Rubin Likelihood Ratio Test *p*<0.001), including “Normal” class (intercept= 0.838, *p*<0.001; linear slope= -0.193, *p*<0.001; quadratic slope= 0.339, *p*<0.001), “Progressive” class (intercept= 2.204, *p*<0.001; linear slope= 6.395, *p*<0.001; quadratic slope= -0.818, *p*<0.001), and “High-onset” class (intercept= 9.795, *p*<0.001; linear slope= -1.296, *p*<0.001; quadratic slope= 0.584, *p*<0.001). It showed similar pattern of disability trajectory classes with our analytic sample (n= 4,149).


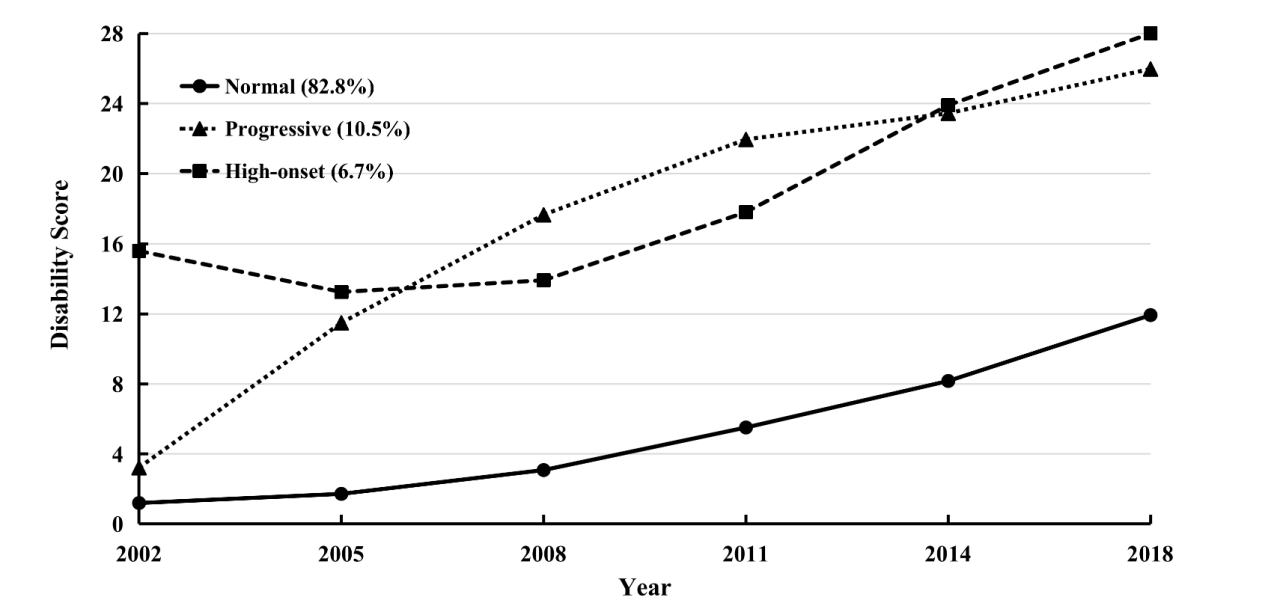


**Supplementary Fig. 4 |** Heterogenous disability trajectory classes of older adults with adjusting time-varying covariates (age) and time invariant covariates (sex, and education) for at least three waves (n= 4,149). Disability score (0-28) was measured by the sum score of BADL (0-12) and IADL (0-16). Three-class GMM was selected as final model (Bayesian information criteria= 97,625.671; entropy= 0.942; Vuong-Lo-Mendell-Rubin Likelihood Ratio Test *p*<0.001), including “Normal” class, “Progressive” class, and “High-onset” class. It showed similar pattern of disability trajectory classes with our analytic sample (n= 4,149).


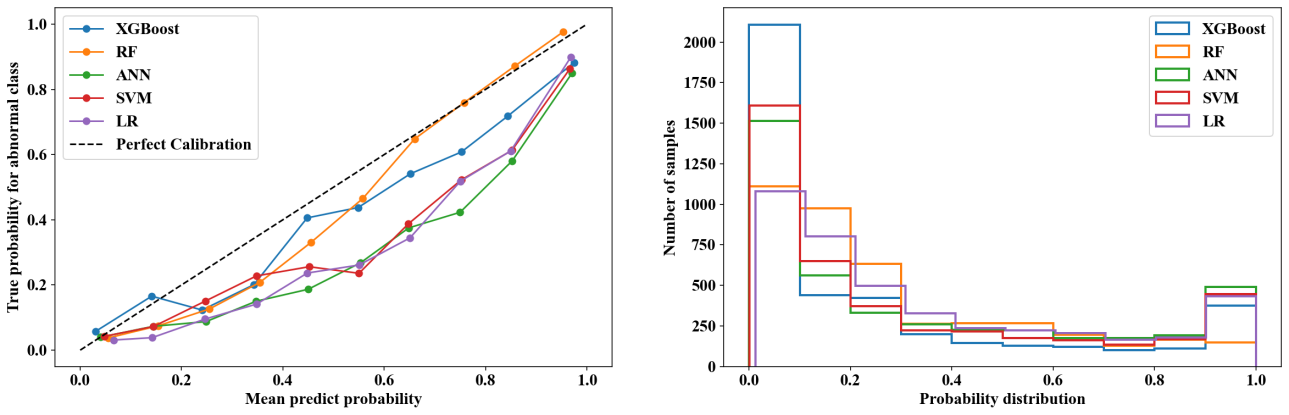


**Supplementary Fig. 5 |** Reliability curve and histogram of prediction probability of the models based on nested cross-validation.


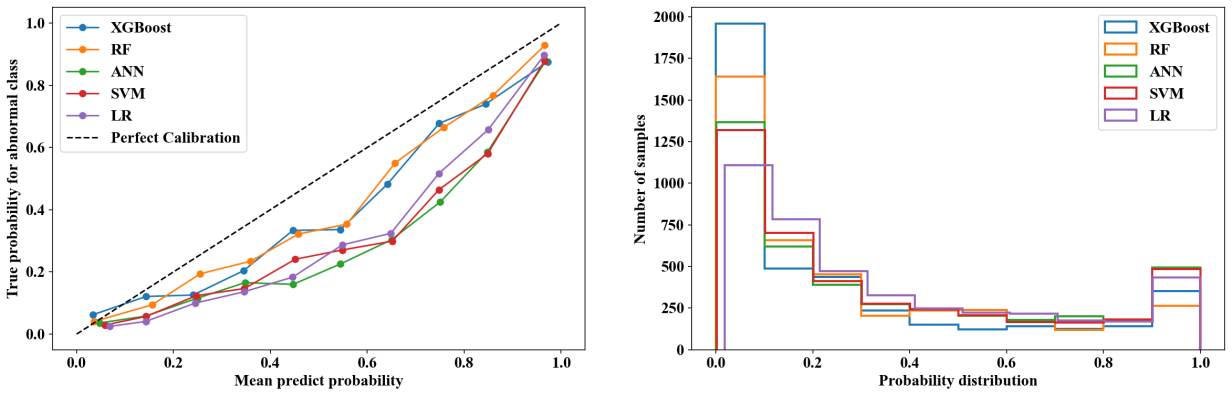


**Supplementary Fig. 6 |** Reliability curve and histogram of prediction probability of the models based on nested cross-validation when LASSO feature selection is performed.


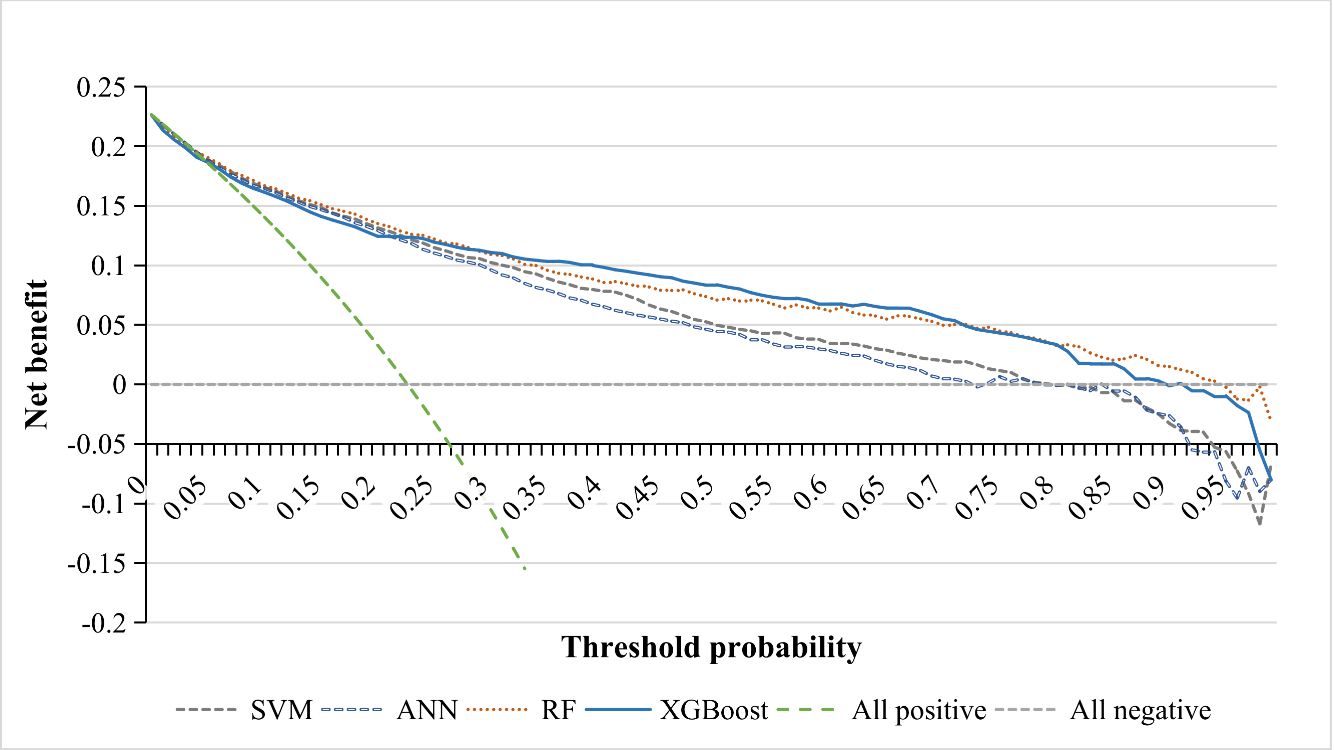


**Supplementary Fig. 7 |** Decision curve analysis for the prediction models when LASSO feature selection is performed.

**
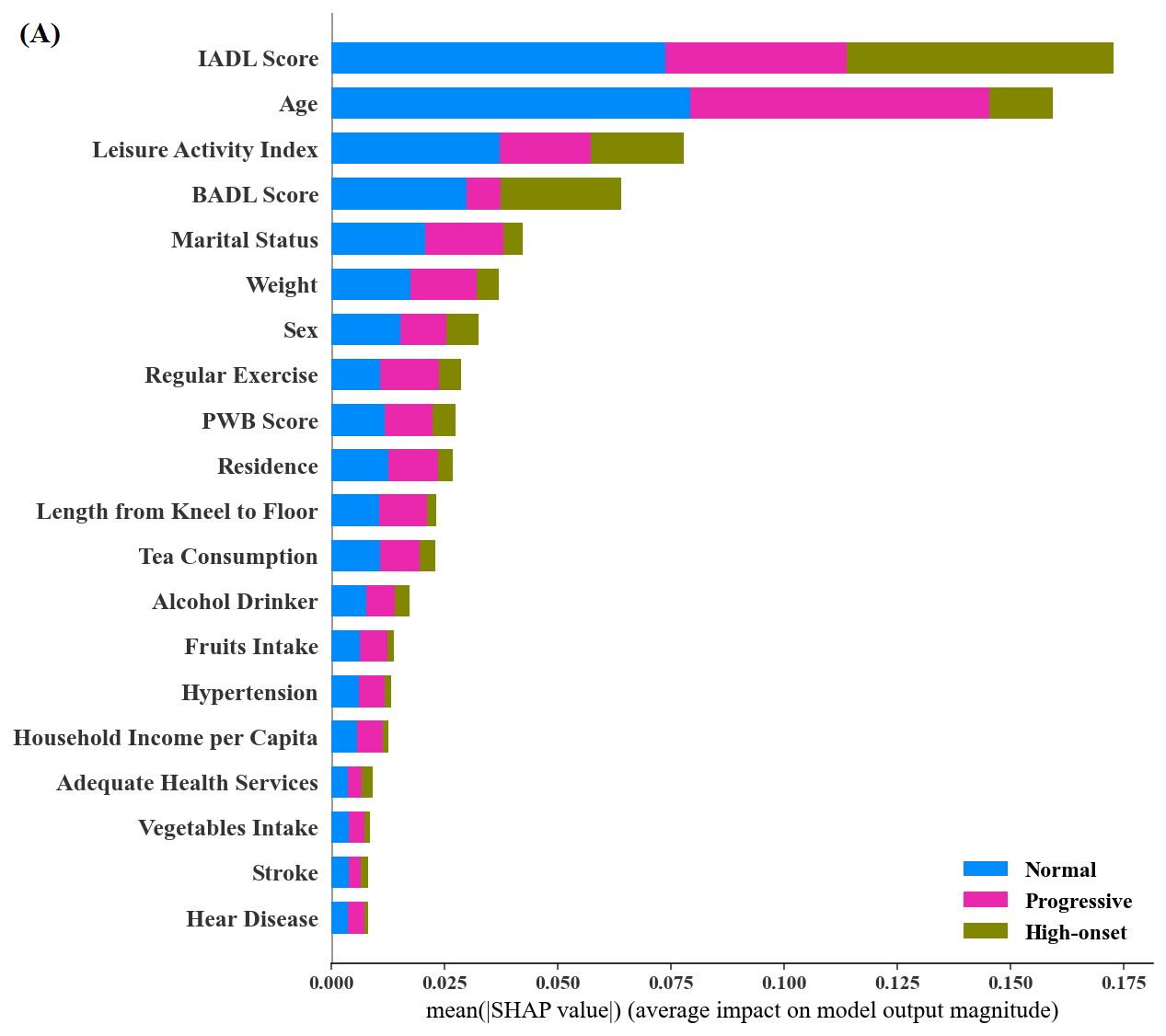

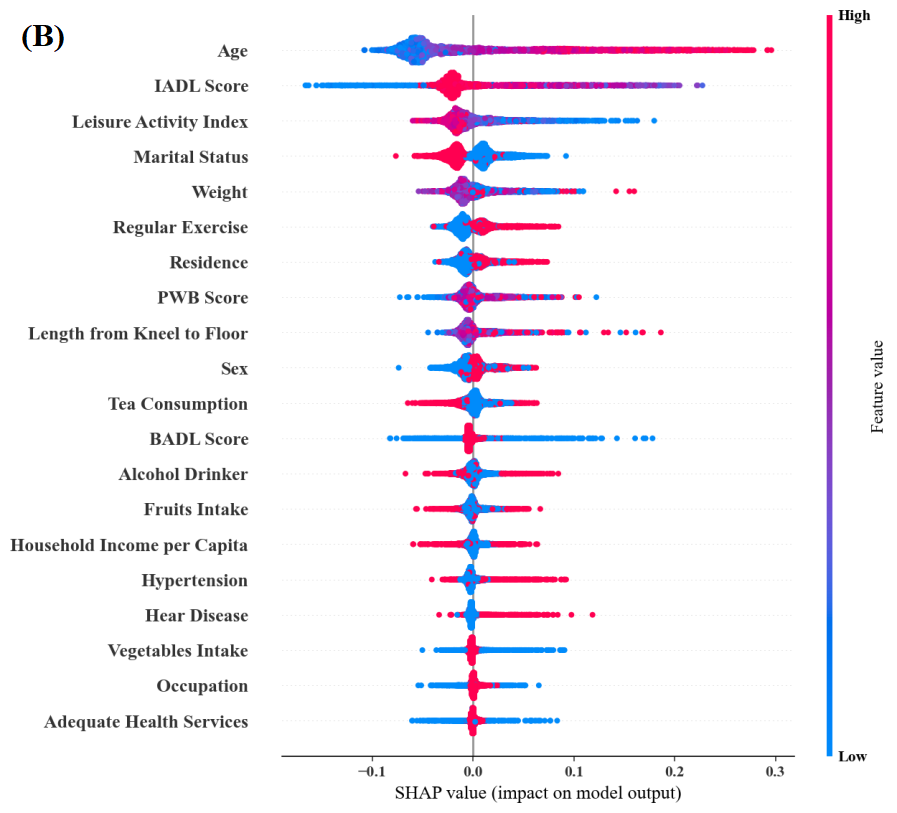
**

**
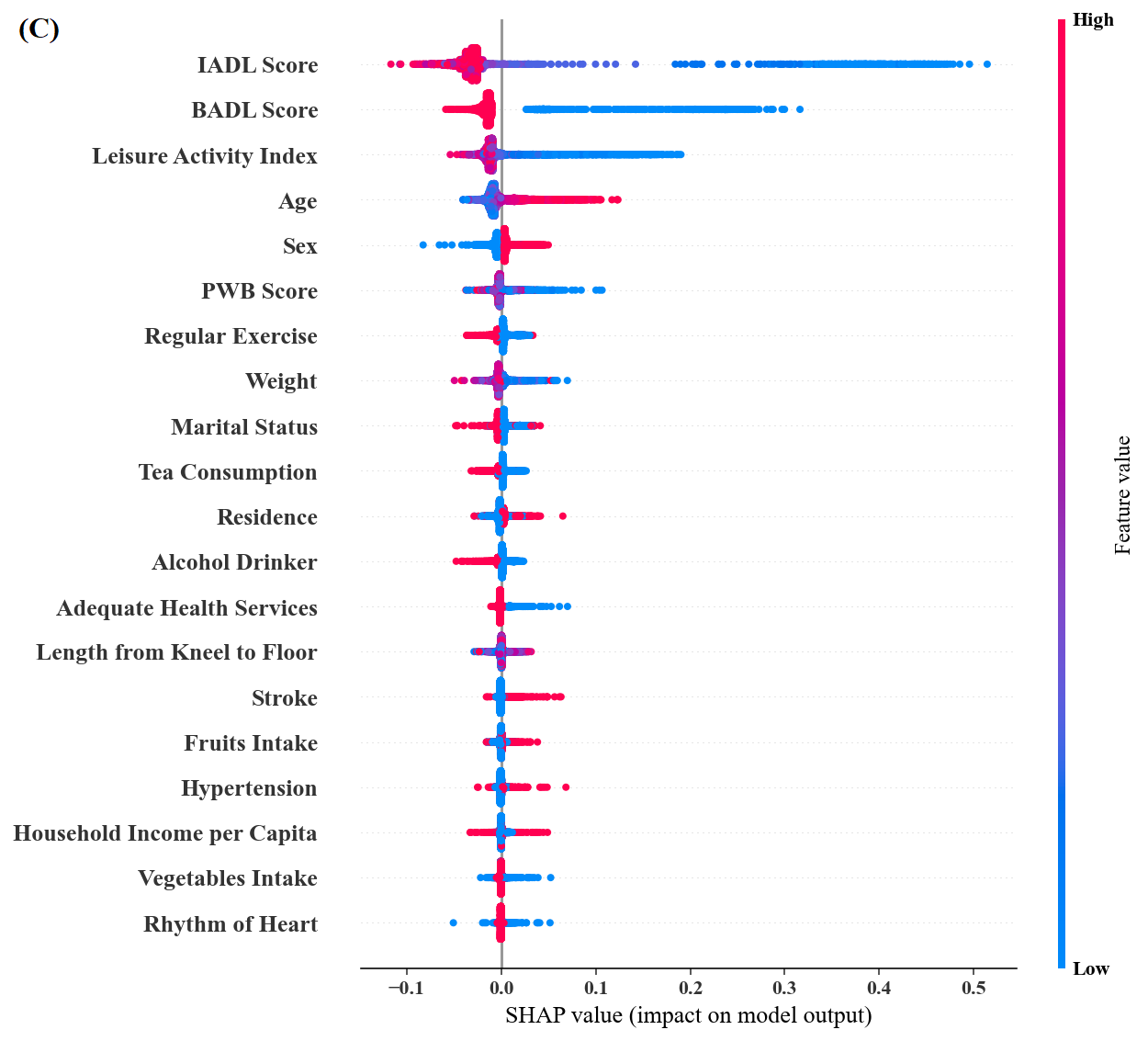

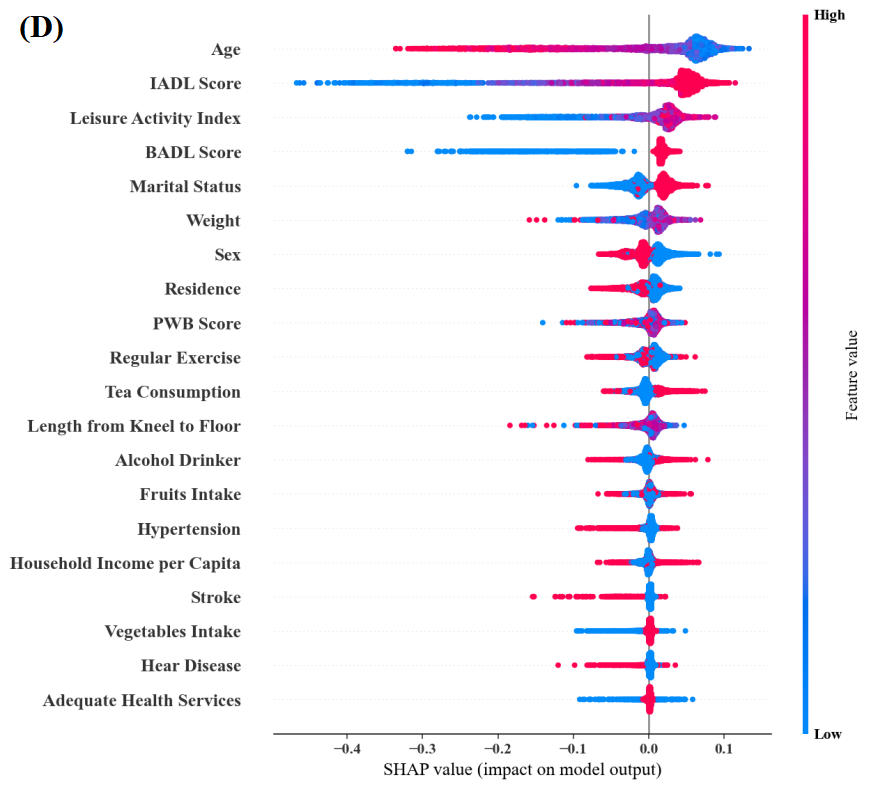
**

**Supplementary Fig. 8 |** The relative feature importance (top 20) of RF in three-class prediction when LASSO feature selection is performed. A: overall feature importance; B: SHAP summary plot when the expected trajectory is Progressive; C: SHAP summary plot when the expected trajectory is High-onset; D: SHAP summary plot when the expected trajectory is Normal.

**
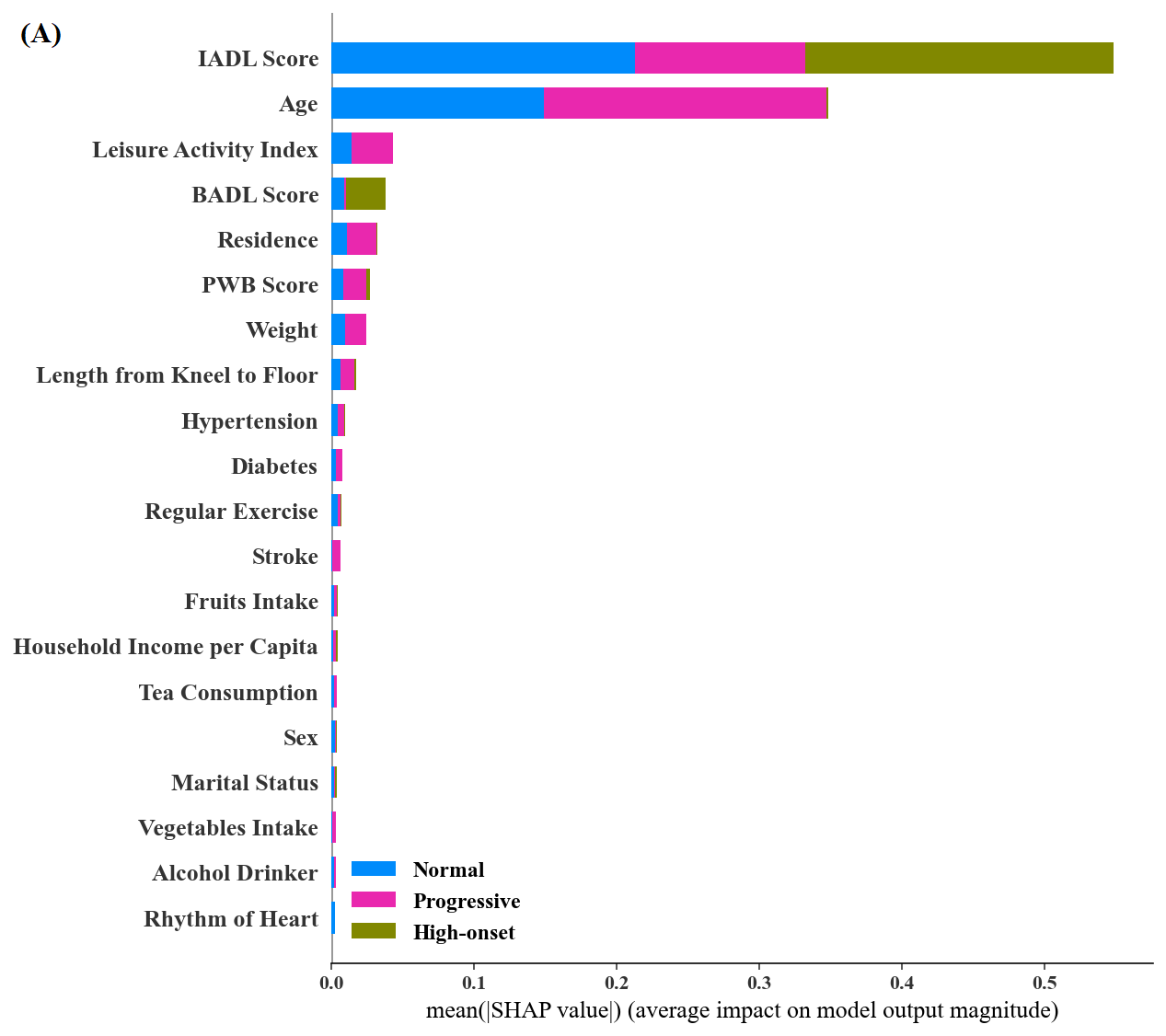

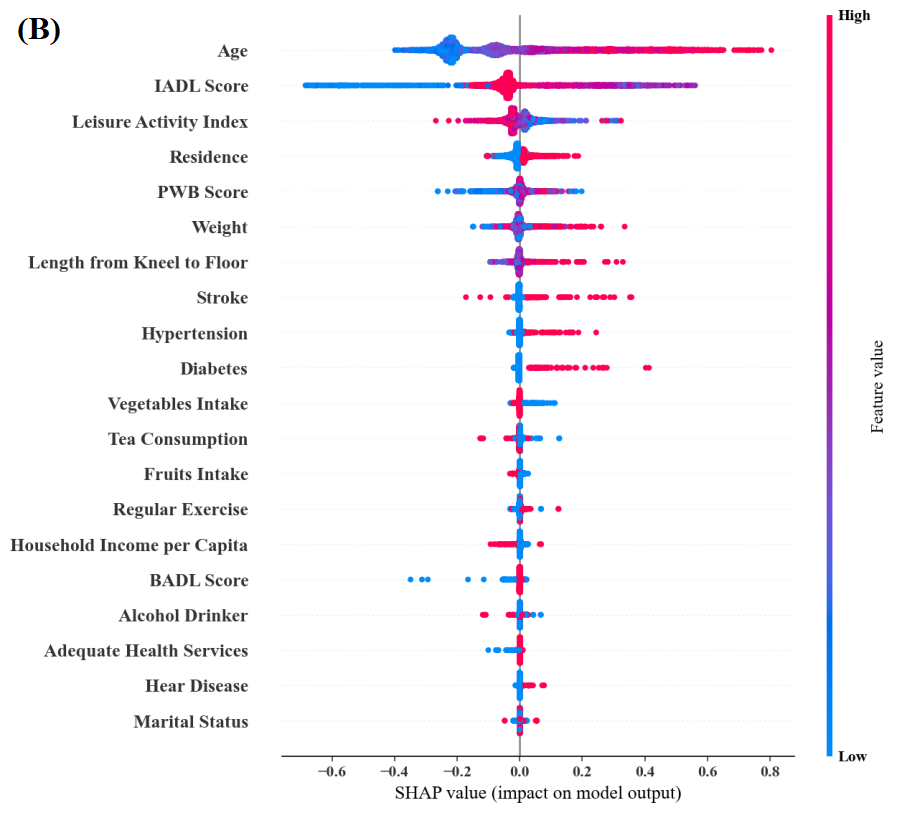
**

**
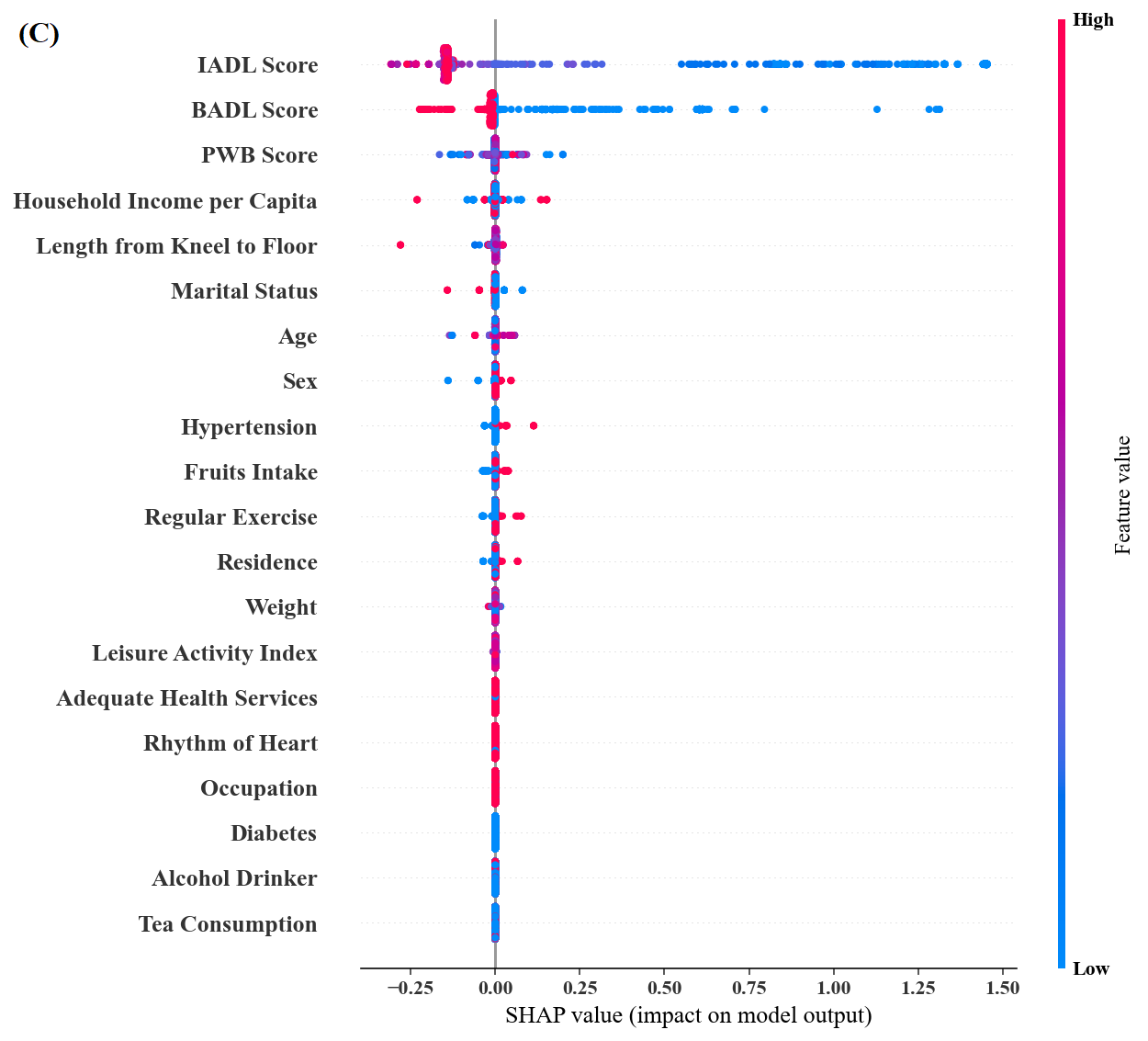

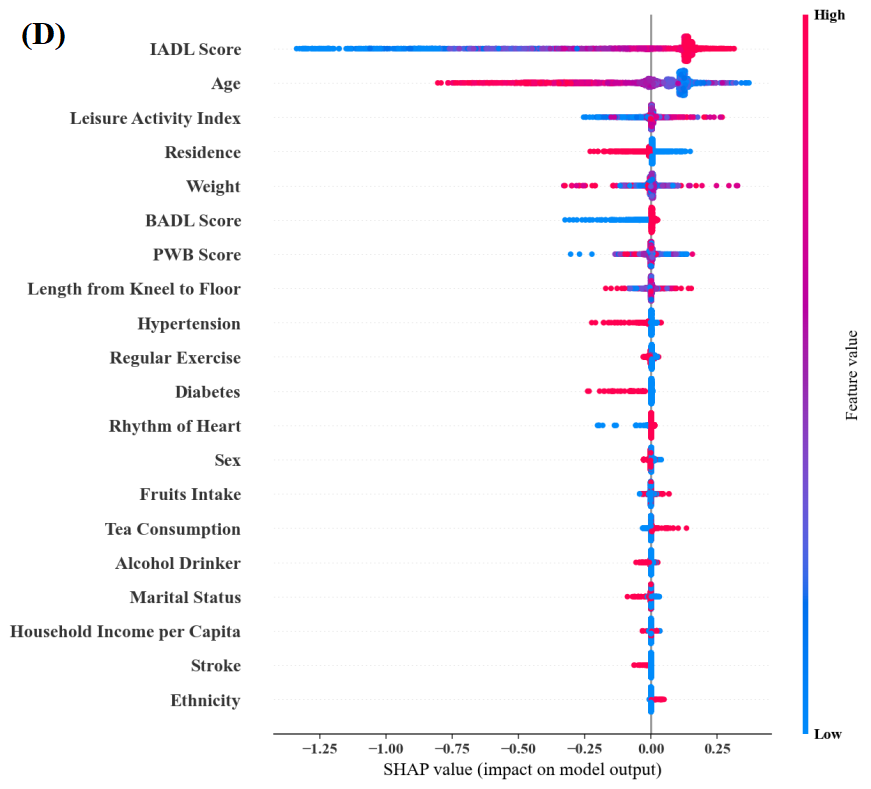
**

**Supplementary Fig. 9 |** The relative feature importance (top 20) of XGBoost in three-class prediction when LASSO feature selection is performed. A: overall feature importance; B: SHAP summary plot when the expected trajectory is Progressive; C: SHAP summary plot when the expected trajectory is High-onset; D: SHAP summary plot when the expected trajectory is Normal.

**
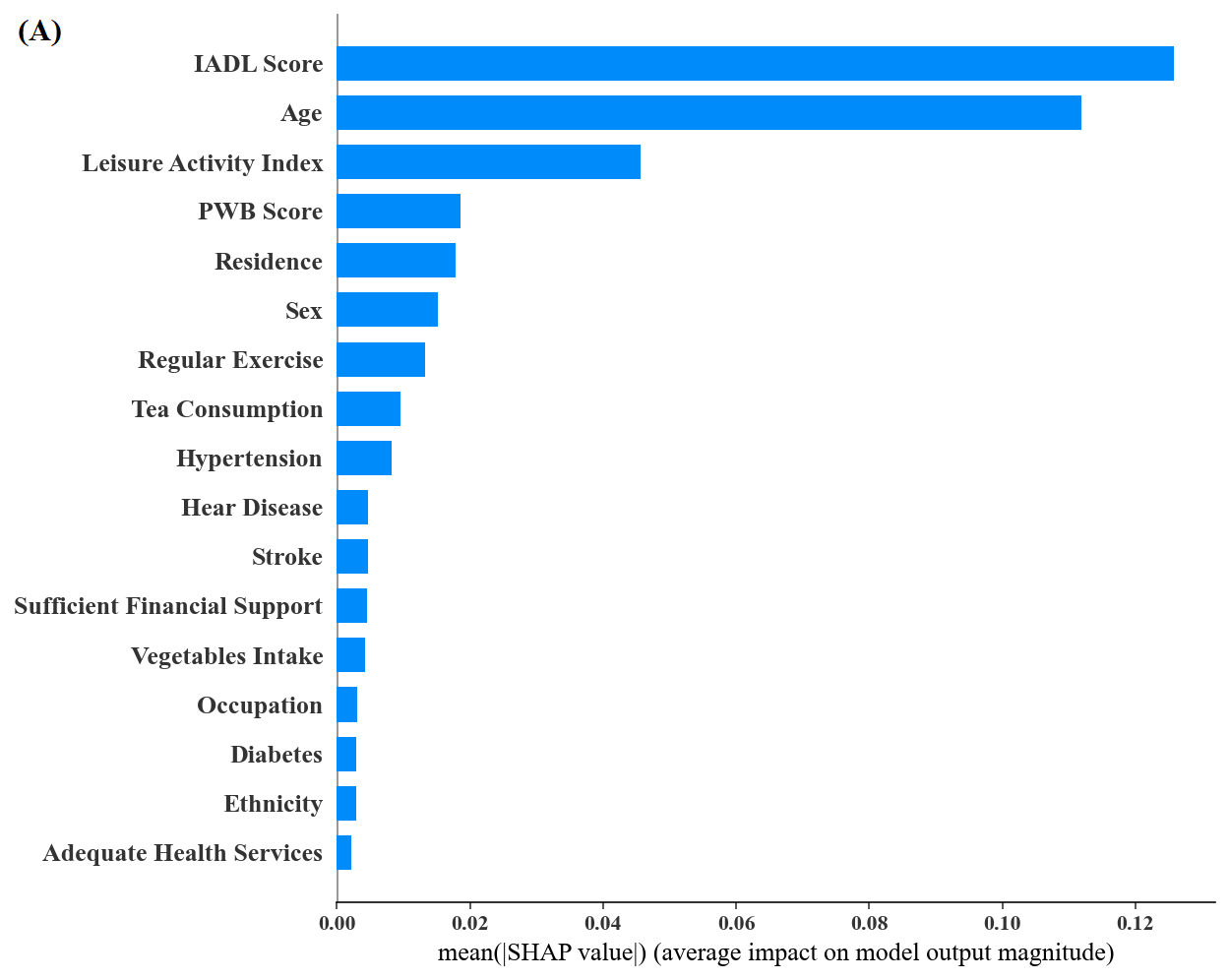

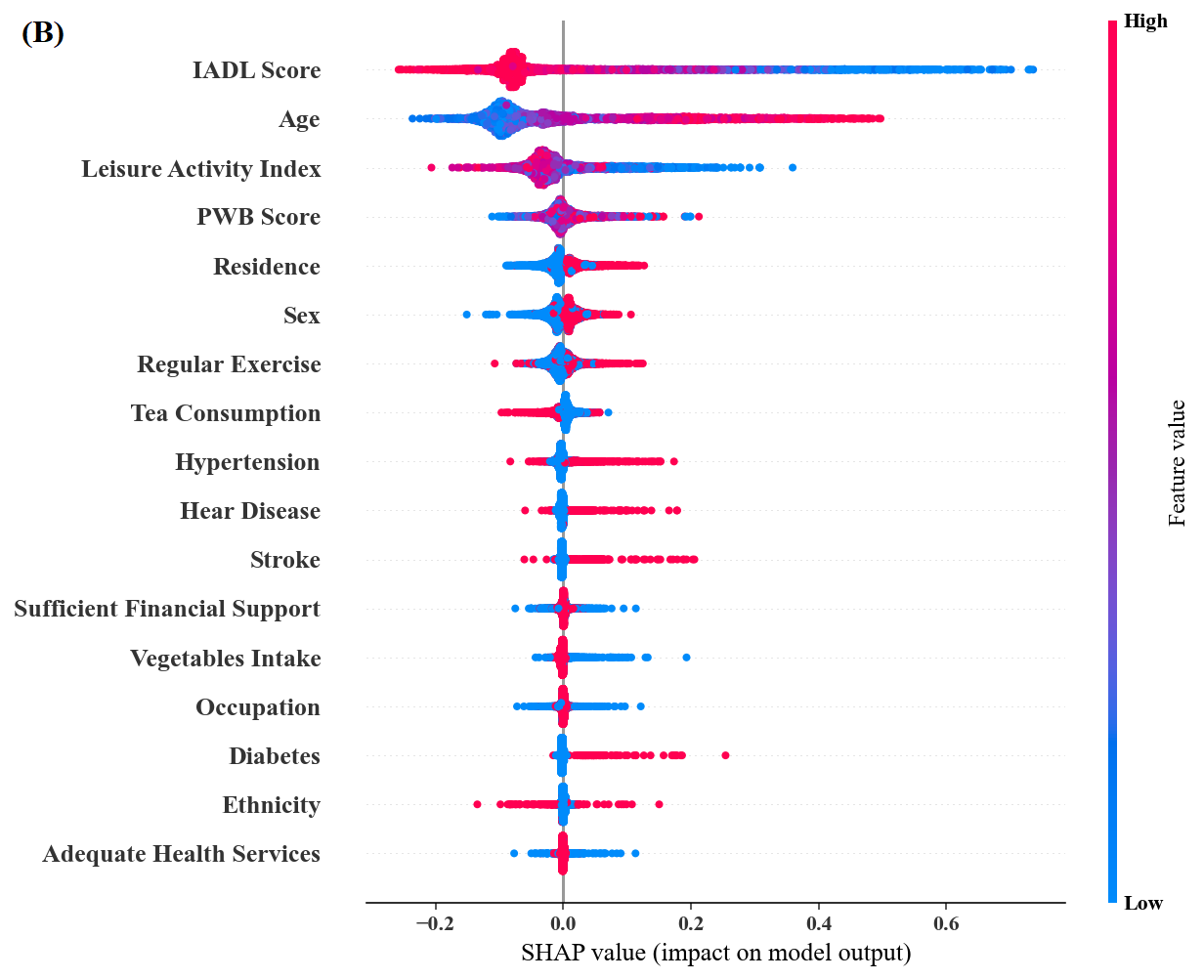
**

**Supplementary Fig. 10 |** The relative feature importance of RF in two-class prediction when LASSO feature selection is performed. A: overall feature importance; B: SHAP summary plot when the expected outcome is Abnormal.

**
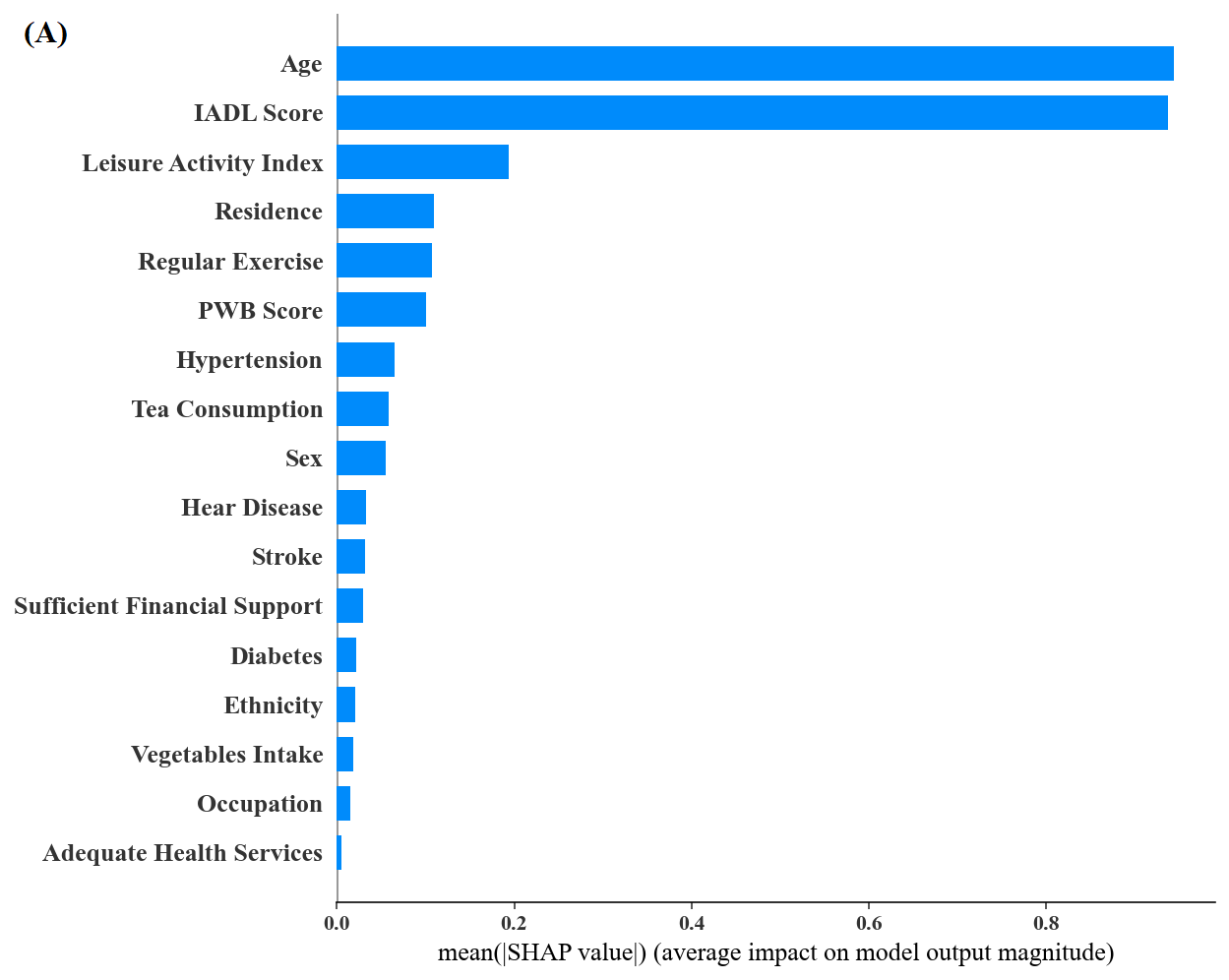

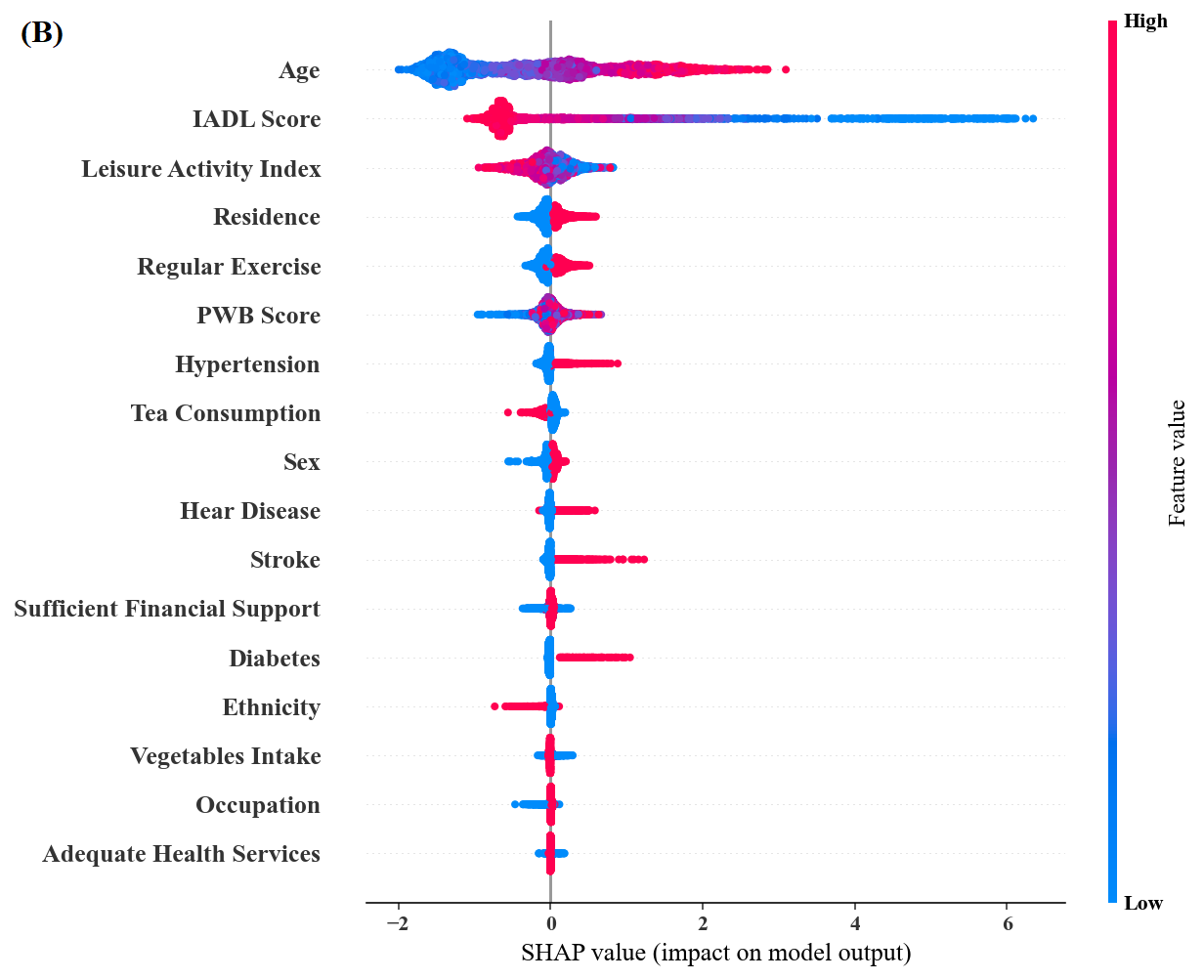
**

**Supplementary Fig. 11 |** The relative feature importance of XGBoost in two-class prediction when LASSO feature selection is performed. A: overall feature importance; B: SHAP summary plot when the expected outcome is Abnormal.

**
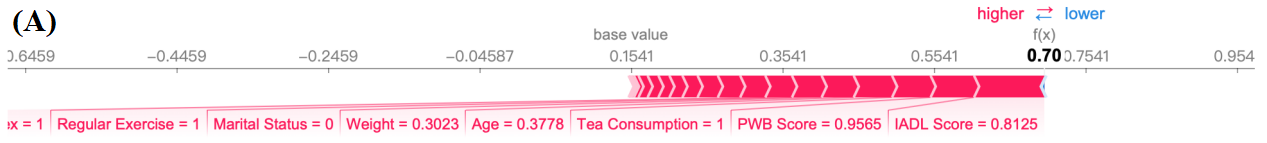

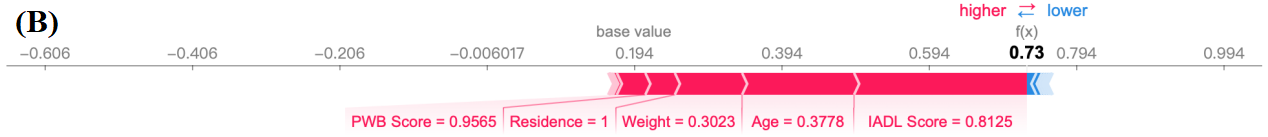

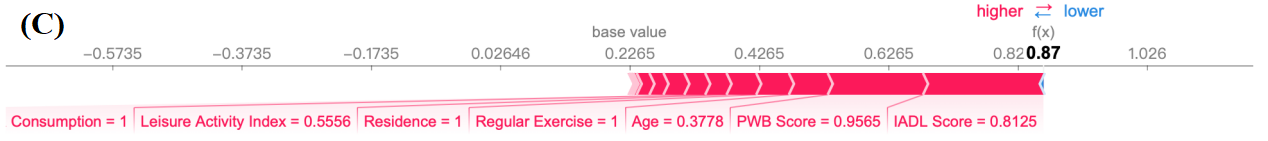

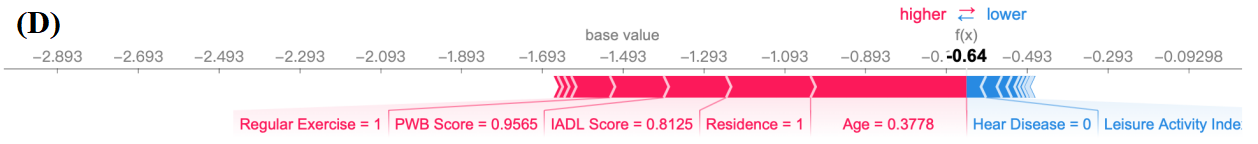
**

**Supplementary Fig. 12 |** Local interpretation of samples based on RF (A, C) and XGBoost (B, D) in LASSO selection data set. A: RF explanation when the expected trajectory is progressive; B: XGBoost explanation when the expected trajectory is progressive; C: RF explanation when the expected trajectory is abnormal; D: XGBoost explanation when the expected trajectory is abnormal.

**Supplementary Table 1 |** Measurement of variables in 2002.

| **Variables** | **Measurement** |
| --- | --- |
| **Sociodemographic Characteristics** | |
| Age | Continuous Variable |
| Sex | 0=Male, 1=Female |
| Ethnicity | 0=Han Ancestry, 1=Minority |
| Education | 0=Illiterate, 1=Literate |
| Occupation | 0=Low level, 1=High level |
| Marital Status | 0=Unmarried/Separated/Divorced/Widowed;  1=Married |
| Residence | 0=Rural, 1=Urban |
| Co-residence | 0=Living alone, 1=With family |
| **Lifestyles** |  |
| Fruits Intake | 0=Low frequency, 1=High frequency |
| Vegetables Intake | 0=Low frequency, 1=High frequency |
| Tea Consumption | 0=Low frequency, 1=High frequency |
| Smoker | 0=No, 1=Yes |
| Alcohol Drinker | 0=No, 1=Yes |
| Regular Exercise | 0=No, 1=Yes |
| Leisure Activity Index | Continuous Variable |
| **Objective Examination** |  |
| Weight | Continuous Variable |
| Systolic Pressure | Continuous Variable |
| Diastolic Pressure | Continuous Variable |
| Rhythm of Heart | 0=Irregular, 1=Regular |
| Heart Rate | Continuous Variable |
| Length from Wrist to Shoulder | Continuous Variable |
| Length from Kneel to Floor | Continuous Variable |
| **Mental & Cognitive &Physical State** | |
| PWB Score* | Continuous Variable |
| MMSE Score* | Continuous Variable |
| BADL Score* | Continuous Variable |
| IADL Score* | Continuous Variable |
| Chronic Condition | Continuous Variable |
| Hypertension | 0=No, 1=Yes |
| Diabetes | 0=No, 1=Yes |
| Stroke | 0=No, 1=Yes |
| Hear Disease | 0=No, 1=Yes |
| **Family Socioeconomic factors** |  |
| Household Income per Capita | 0=Low level, 1=High level |
| Adequate Health Services | 0=No, 1=Yes |
| Sufficient Financial Support | 0=No, 1=Yes |

Abbreviations: PWB, psychological well-being; BADL, basic activity of daily living; IADL, instrumental activity of daily living; MMSE, Mini-Mental State Examination.

**Supplementary Table 2 |** The parameters of disability trajectories with consideration of covariates (Age, Sex, Education).

|  |  | Coefficients | *P* value |
| --- | --- | --- | --- |
| Sex | Intercept | 0.584 | <0.001 |
|  | Linear slope | 0.027 | 0.836 |
|  | Quadratic slope | 0.014 | 0.732 |
| Education | Intercept | -0.328 | <0.001 |
|  | Linear slope | 0.064 | 0.622 |
|  | Quadratic slope | -0.046 | 0.243 |
| Age2002^a^ | **—** | 0.121 | <0.001 |
| Age2005 | **—** | 0.169 | <0.001 |
| Age2008 | **—** | 0.256 | <0.001 |
| Age2011 | **—** | 0.381 | <0.001 |
| Age2014 | **—** | 0.531 | <0.001 |
| Age2018 | **—** | 0.721 | <0.001 |

^a^ Age2002 represents the participants’ age in wave 2002, the others were in similar coding ways.

**Supplementary Table 3 |** Comparison of sample characteristics between analytical sample and drop-out sample

|  | **Multivariate Logistic** | |
| --- | --- | --- |
|  | OR | P-value |
| Age | 0.94 (0.93-0.95) | 0.000 |
| Sex (Ref: Male) |  |  |
| Female | 1.18 (1.00-1.39) | 0.044 |
| Ethnicity (Ref: Han Ancestry) |  |  |
| Minority | 1.23 (0.94-1.62) | 0.135 |
| Education (Ref: Illiterate) |  |  |
| Literate | 0.96 (0.83-1.11) | 0.575 |
| Occupation (Ref: Low level) |  |  |
| High level | 1.10 (0.93-1.31) | 0.282 |
| Marital Status  (Ref:Unmarried/Separated/Divorced/Widowed) |  |  |
| Married | 0.99 (0.86-1.15) | 0.907 |
| Residence (Ref: Rural) |  |  |
| Urban | 0.65 (0.57-0.74) | 0.000 |
| Co-residence (Ref: Living alone) |  |  |
| With family | 1.37 (1.13-1.65) | 0.001 |
| Fruit Intake (Ref: Low frequency) |  |  |
| High frequency | 0.86 (0.75-0.98) | 0.022 |
| Vegetables Intake  (Ref: Low frequency) |  |  |
| High frequency | 1.04 (0.84-1.28) | 0.750 |
| Tea Consumption (Ref: Low frequency) |  |  |
| High frequency | 0.90 (0.79-1.03) | 0.115 |
| Smoker (Ref: No) |  |  |
| Yes | 1.01 (0.88-1.17) | 0.854 |
| Alcohol Drinker (Ref: No) |  |  |
| Yes | 1.07 (0.93-1.24) | 0.347 |
| Regular Exercise (Ref: No) |  |  |
| Yes | 1.04 (0.91-1.18) | 0.611 |
| Leisure Activity Index | 2.07 (1.17-3.64) | 0.012 |
| Weight | 1.00 (0.99-1.01) | 0.888 |
| Systolic Pressure | 1.00 (0.99-1.00) | 0.106 |
| Diastolic Pressure | 1.00 (0.99-1.00) | 0.296 |
| Rhythm of Heart (Ref: Irregular) |  |  |
| Regular | 1.16 (0.90-1.49) | 0.255 |
| Heart Rate | 0.99 (0.98-0.99) | 0.000 |
| Length from Wrist to Shoulder | 0.99 (0.97-1.00) | 0.067 |
| Length from Kneel to Floor | 1.01 (0.99-1.03) | 0.217 |
| PWB Score* | 1.01 (0.99-1.03) | 0.292 |
| BADL Score* | 1.03 (0.93-1.15) | 0.576 |
| IADL Score* | 1.09 (1.07-1.13) | 0.000 |
| MMSE score* | 1.02 (1.00-1.04) | 0.101 |
| Chronic Condition | 0.90 (0.82-1.00) | 0.039 |
| Hypertension (Ref: No) |  |  |
| Yes | 1.34 (1.08-1.65) | 0.007 |
| Diabetes (Ref: No) |  |  |
| Yes | 0.78 (0.54-1.14) | 0.196 |
| Stroke (Ref: No) |  |  |
| Yes | 0.98 (0.73-1.33) | 0.915 |
| Hear Disease (Ref: No) |  |  |
| Yes | 1.21 (0.96-1.52) | 0.108 |
| Household Income per Capita (Ref: Low leavel) |  |  |
| High level | 0.76 (0.66-0.88) | 0.000 |
| Adequate Health Services (Ref: No) |  |  |
| Yes | 1.09 (0.84-1.42) | 0.497 |
| Sufficient Financial Support (Ref: No) |  |  |
| Yes | 1.00 (0.84-1.20) | 0.972 |

The numbers of analytical sample and drop-out sample were 4,149 and 11,915 respectively. Multivariate logistic models were used, and the drop-out sample was set as the reference.

Abbreviations: OR, odds ratio; Ref, reference; PWB, psychological well-being; BADL, basic activity of daily living; IADL, instrumental activity of daily living; MMSE, Mini-Mental State Examination.

**Supplementary Table 4 |** Selected variables in each imputation data with LASSO for three-class prediction task.

| variables | M1 | M2 | M3 | M4 | M5 |
| --- | --- | --- | --- | --- | --- |
| Age | √ | √ | √ | √ | √ |
| Sex | √ | √ | √ | √ | √ |
| Ethnicity | √ | √ | √ | √ | √ |
| Education | × | × | √ | × | √ |
| Occupation | √ | √ | √ | √ | √ |
| Marital Status | √ | √ | √ | √ | √ |
| Residence | √ | √ | √ | √ | √ |
| Co-residence | × | × | √ | √ | × |
| Fruit Intake | √ | √ | √ | √ | √ |
| Vegetables Intake | √ | √ | √ | √ | √ |
| Tea Consumption | √ | √ | √ | √ | √ |
| Smoker | × | × | × | × | × |
| Alcohol Drinker | √ | √ | √ | √ | √ |
| Regular Exercise | √ | √ | √ | √ | √ |
| Leisure Activity Index | √ | √ | √ | √ | √ |
| Weight | √ | √ | √ | √ | √ |
| Systolic Pressure | × | × | × | × | × |
| Diastolic Pressure | × | × | × | × | × |
| Rhythm of Heart | √ | √ | √ | √ | √ |
| Heart Rate | × | × | × | × | × |
| Length from Wrist to Shoulder | × | × | × | × | × |
| Length from Kneel to Floor | √ | √ | √ | √ | √ |
| PWB Score* | √ | √ | √ | √ | √ |
| BADL Score* | √ | √ | √ | √ | √ |
| IADL Score* | √ | √ | √ | √ | √ |
| MMSE score* | × | × | × | × | × |
| Chronic Condition | × | × | × | × | × |
| Hypertension | √ | √ | √ | √ | √ |
| Diabetes | √ | √ | √ | √ | √ |
| Stroke | √ | √ | √ | √ | √ |
| Hear Disease | √ | √ | √ | √ | √ |
| Household Income per Capita | √ | √ | √ | √ | × |
| Adequate Health Services | √ | √ | √ | √ | √ |
| Sufficient Financial Support | × | × | × | × | × |

Note: M1-M5 represent the five sets of data obtained by multiple imputation according to the original data. “√” means the variables selected by LASSO and “×” represents the variables not included by LASSO in every imputation data.

**Supplementary Table 5 |** Optimal hyper-parameters for a set of imputation data using nested cross-validation in three-class prediction task.

| Fold | **LR** | **SVM** | **RF** | **ANN** | **XGboost** |
| --- | --- | --- | --- | --- | --- |
| 1 | Penalty:“L2”  C= 0.1 | Kernel: “rbf  Gamma=0.1  C=100 | n_estimators=60  max_features=3 | hidden_layer_sizes:  [10, 10] | learning_rate=0.01  Gamma=0.001  max_depth=5 |
| 2 | Penalty:“L2”  C= 0.1 | Kernel: “rbf”  Gamma=0.1  C=10 | n_estimators=110  max_features=4 | hidden_layer_sizes:  [10] | learning_rate=0.01  Gamma=1  max_depth=5 |
| 3 | Penalty:“L2”  C= 0.1 | Kernel: “rbf”  Gamma=0.1  C=100 | n_estimators=70  max_features=7 | hidden_layer_sizes:  [10, 5, 10] | learning_rate=0.01  Gamma=0.01  max_depth=6 |
| 4 | Penalty:“L1”  C= 10 | Kernel: “rbf”  Gamma=0.1  C=1 | n_estimators=130  max_features=2 | hidden_layer_sizes:  [5, 5, 5] | learning_rate=0.05  Gamma=0.001  max_depth=6 |
| 5 | Penalty:“L2”  C= 1 | Kernel: “rbf”  Gamma=0.1  C=10 | n_estimators=80  max_features=6 | hidden_layer_sizes:  [10, 5] | learning_rate=0.1  Gamma=1  max_depth=4 |
| 6 | Penalty:“L2”  C= 1 | Kernel:“rbf”  Gamma=0.1  C=10 | n_estimators=100  max_features=2 | hidden_layer_sizes:  [5, 10] | learning_rate=0.01  Gamma=1  max_depth=10 |
| 7 | Penalty:“L2”  C= 10 | Kernel: “rbf”  Gamma=0.1  C=10 | n_estimators=110  max_features=2 | hidden_layer_sizes:  [10, 5,10] | learning_rate=1  Gamma=0.01  max_depth=8 |
| 8 | Penalty:“L1”  C= 0.1 | Kernel:“rbf”  Gamma=0.1  C=10 | n_estimators=60  max_features=7 | hidden_layer_sizes:  [10, 5, 10] | learning_rate=0.01  Gamma=1  max_depth=8 |
| 9 | Penalty:“L2”  C= 10 | Kernel: “rbf”  Gamma=0.1  C=10 | n_estimators=30  max_features=5 | hidden_layer_sizes:  [10, 5, 10] | learning_rate=0.01  Gamma=0.01  max_depth=8 |
| 10 | Penalty:“L2”  C= 0.1 | Kernel: “rbf”  Gamma=1  C=0.1 | n_estimators=40  max_features=2 | hidden_layer_sizes:  [5, 5, 5] | learning_rate=0.1  Gamma=0.001  max_depth=8 |

For LR, the “penalty” was tuned from “none”, “L1” and “L2”; the “C” parameter is selected from 0.001, 0.1, 1, 10, 100. For SVM, the “kernel” parameter is searching from “linear” and “rbf”; the “gamma” parameters include 0.001, 0.1, 1, 10, 100; the “C” parameter is selected from 0.001, 0.1, 1, 10, 100. For RF, the “n_estimators” parameter is from 10 to 150 by 10; the “max_features” parameter is from 1 to 12. For ANN, the “hidden_layer_sizes” parameter is selected from “[5,5]”, “[10,10]”, “[5,10]”, “[10,5]”, “[10,10,10]”, “[5,5,5]”, “[5,10,10]”, “[10,5,10]”, “[5,10,5]” “[10,10,5]”, “[5,5,10]”, “[10,5,5]”. For XGboost, the “learning_rate” parameters include 0.01, 0.05, 0.1, 0.5, 1; the “gamma” parameter is selected from 0.01, 0.1, 1, 10; the “max_depth” parameter is from 1 to 10. All models’ hyper-parameters except the above are set to default value.

**Supplementary Table 6 |** Performance of machine learning algorithms for trajectories prediction^a^.

|  | **Full variables** | | | | | **Selected variables with LASSO** | | | | |
| --- | --- | --- | --- | --- | --- | --- | --- | --- | --- | --- |
|  | **LR** | **SVM** | **RF** | **ANN** | **XGBoost** | **LR** | **SVM** | **RF** | **ANN** | **XGBoost** |
| Accuracy | 0.687 | 0.719 | 0.768 | 0.704 | 0.764 | 0.702 | 0.707 | 0.759 | 0.700 | 0.737 |
| Recall | 0.774 | 0.860 | 0.896 | 0.775 | 0.874 | 0.783 | 0.839 | 0.891 | 0.774 | 0.879 |
| Precision | 0.757 | 0.857 | 0.926 | 0.771 | 0.871 | 0.768 | 0.825 | 0.907 | 0.754 | 0.885 |
| F1 Score | 0.735 | 0.857 | 0.909 | 0.744 | 0.868 | 0.745 | 0.827 | 0.898 | 0.734 | 0.880 |
| Hamming | 0.226 | 0.140 | 0.104 | 0.225 | 0.126 | 0.217 | 0.161 | 0.109 | 0.226 | 0.121 |
| Jaccard | 0.623 | 0.781 | 0.853 | 0.636 | 0.798 | 0.634 | 0.742 | 0.839 | 0.623 | 0.814 |
| Kappa | 0.408 | 0.464 | 0.543 | 0.341 | 0.554 | 0.424 | 0.439 | 0.549 | 0.401 | 0.533 |

^a^ Trajectory classes were identified among older adults with complete information for at least four waves (n= 2,457).

Note: accuracy, recall, precision, F1 score were all calculated with weighted metrics. Hamming, Jaccard, and Kappa refer to Hamming distance, Jaccard similarity coefficient, and Cohen's kappa score.

**Supplementary Table 7 |** Performance of machine learning algorithms for trajectories prediction^a^.

|  | **Full variables** | | | | | **Selected variables with LASSO** | | | | |
| --- | --- | --- | --- | --- | --- | --- | --- | --- | --- | --- |
|  | **LR** | **SVM** | **RF** | **ANN** | **XGBoost** | **LR** | **SVM** | **RF** | **ANN** | **XGBoost** |
| Accuracy | 0.760 | 0.743 | 0.777 | 0.755 | 0.771 | 0.755 | 0.745 | 0.779 | 0.755 | 0.777 |
| Recall | 0.779 | 0.782 | 0.815 | 0.776 | 0.810 | 0.775 | 0.781 | 0.816 | 0.776 | 0.817 |
| Precision | 0.775 | 0.782 | 0.821 | 0.768 | 0.812 | 0.771 | 0.779 | 0.823 | 0.768 | 0.825 |
| F1 Score | 0.765 | 0.780 | 0.818 | 0.763 | 0.810 | 0.761 | 0.778 | 0.819 | 0.763 | 0.820 |
| Hamming | 0.221 | 0.218 | 0.185 | 0.224 | 0.190 | 0.225 | 0.219 | 0.184 | 0.224 | 0.183 |
| Jaccard | 0.639 | 0.664 | 0.712 | 0.638 | 0.701 | 0.634 | 0.662 | 0.714 | 0.638 | 0.715 |
| Kappa | 0.565 | 0.520 | 0.585 | 0.554 | 0.581 | 0.559 | 0.522 | 0.587 | 0.554 | 0.589 |

^a^Trajectory classes were identified among older adults with complete information of IADL for at least three waves (n= 4,149).

Note: accuracy, recall, precision, F1 score were all calculated with weighted metrics. Hamming, Jaccard, and Kappa refer to Hamming distance, Jaccard similarity coefficient, and Cohen's kappa score.

**Supplementary Table 8 |** Performance of machine learning algorithms for trajectories prediction^a^.

|  | **Full variables** | | | | | **Selected variables with LASSO** | | | | |
| --- | --- | --- | --- | --- | --- | --- | --- | --- | --- | --- |
|  | **LR** | **SVM** | **RF** | **ANN** | **XGBoost** | **LR** | **SVM** | **RF** | **ANN** | **XGBoost** |
| Accuracy | 0.653 | 0.653 | 0.695 | 0.679 | 0.716 | 0.652 | 0.641 | 0.684 | 0.655 | 0.692 |
| Recall | 0.729 | 0.836 | 0.870 | 0.742 | 0.853 | 0.742 | 0.805 | 0.862 | 0.743 | 0.863 |
| Precision | 0.709 | 0.843 | 0.911 | 0.722 | 0.857 | 0.712 | 0.785 | 0.891 | 0.714 | 0.891 |
| F1 Score | 0.685 | 0.838 | 0.888 | 0.705 | 0.852 | 0.701 | 0.791 | 0.875 | 0.703 | 0.875 |
| Hamming | 0.271 | 0.164 | 0.130 | 0.258 | 0.147 | 0.258 | 0.195 | 0.138 | 0.257 | 0.137 |
| Jaccard | 0.561 | 0.755 | 0.822 | 0.583 | 0.774 | 0.583 | 0.696 | 0.805 | 0.586 | 0.804 |
| Kappa | 0.371 | 0.430 | 0.500 | 0.408 | 0.510 | 0.376 | 0.413 | 0.487 | 0.376 | 0.498 |

^a^Trajectory classes were identified with consideration of covariates (age, sex, education) among older adults with complete information for at least three waves (n= 4,149).

Note: accuracy, recall, precision, F1 score were all calculated with weighted metrics. Hamming, Jaccard, and Kappa refer to Hamming distance, Jaccard similarity coefficient, and Cohen's kappa score.

**Supplementary Table 9 |** Performance of machine learning algorithms for trajectories prediction^a^.

|  | **Full variables** | | | | | **Selected variables with LASSO** | | | | |
| --- | --- | --- | --- | --- | --- | --- | --- | --- | --- | --- |
|  | **LR** | **SVM** | **RF** | **ANN** | **XGBoost** | **LR** | **SVM** | **RF** | **ANN** | **XGBoost** |
| Accuracy | 0.647 | 0.684 | 0.735 | 0.663 | 0.727 | 0.608 | 0.654 | 0.679 | 0.657 | 0.690 |
| Recall | 0.759 | 0.820 | 0.855 | 0.756 | 0.837 | 0.753 | 0.804 | 0.840 | 0.744 | 0.832 |
| Weighted Precision | 0.747 | 0.821 | 0.882 | 0.733 | 0.838 | 0.740 | 0.786 | 0.853 | 0.734 | 0.830 |
| F1 Score | 0.728 | 0.819 | 0.866 | 0.724 | 0.833 | 0.720 | 0.792 | 0.845 | 0.708 | 0.827 |
| Hamming | 0.241 | 0.180 | 0.145 | 0.244 | 0.163 | 0.247 | 0.196 | 0.160 | 0.256 | 0.168 |
| Jaccard | 0.604 | 0.729 | 0.791 | 0.604 | 0.744 | 0.596 | 0.693 | 0.763 | 0.582 | 0.736 |
| Kappa | 0.366 | 0.326 | 0.394 | 0.340 | 0.421 | 0.355 | 0.341 | 0.376 | 0.341 | 0.423 |

^a^Trajectory classes were identified among older adults with complete information of ADL for at least three waves (n= 4,149), and predictions were made among older adults with complete information of MMSE at baseline (n= 2,469).

Note: accuracy, recall, precision, F1 score were all calculated with weighted metrics. Hamming, Jaccard, and Kappa refer to Hamming distance, Jaccard similarity coefficient, and Cohen's kappa score.

**Supplementary Table 10 |** Selected variables in each imputation data with LASSO for two-class prediction task.

| **Variables** | **M1** | **M2** | **M3** | **M4** | **M5** |
| --- | --- | --- | --- | --- | --- |
| Age | √ | √ | √ | √ | √ |
| Sex | √ | √ | √ | √ | √ |
| Ethnicity | √ | √ | √ | √ | √ |
| Education | × | × | × | × | × |
| Occupation | √ | √ | √ | √ | √ |
| Marital Status | × | × | × | × | × |
| Residence | √ | √ | √ | √ | √ |
| Co-residence | × | × | × | × | × |
| Fruit Intake | × | × | × | × | × |
| Vegetables Intake | √ | √ | √ | √ | √ |
| Tea Consumption | √ | √ | √ | √ | √ |
| Smoker | × | × | × | × | × |
| Alcohol Drinker | × | × | × | × | × |
| Regular Exercise | √ | √ | √ | √ | √ |
| Leisure Activity Index | √ | √ | √ | √ | √ |
| Weight | × | × | × | × | × |
| Systolic Pressure | × | × | × | × | × |
| Diastolic Pressure | × | × | × | × | × |
| Rhythm of Heart | × | × | × | × | × |
| Heart Rate | × | × | × | × | × |
| Length from Wrist to Shoulder | × | × | × | × | × |
| Length from Kneel to Floor | × | × | × | × | × |
| PWB Score* | √ | √ | √ | √ | √ |
| BADL Score* | × | √ | √ | √ | √ |
| IADL Score* | √ | √ | √ | √ | √ |
| MMSE score* | × | × | × | × | × |
| Chronic Condition | × | × | × | × | × |
| Hypertension | √ | √ | √ | √ | √ |
| Diabetes | √ | √ | √ | √ | √ |
| Stroke | √ | √ | √ | √ | √ |
| Hear Disease | √ | √ | √ | √ | √ |
| Household Income per Capita | × | × | × | × | × |
| Adequate Health Services | √ | √ | √ | √ | √ |
| Sufficient Financial Support | √ | √ | √ | √ | √ |

M1-M5 represent the five sets of data obtained by multiple imputation according to the original data. “√” means the variables selected by LASSO and “×” represents the variables not included by LASSO in every imputation data.

**Supplementary Table 11 |** Optimal hyper-parameters for a set of imputation data using nested cross-validation in two-class prediction task.

| Fold | **LR** | **SVM** | **RF** | **ANN** | **XGboost** |
| --- | --- | --- | --- | --- | --- |
| 1 | Penalty:“L2”  C= 1 | Kernel: “rbf  Gamma=0.1  C=1 | n_estimators=90  max_features=4 | hidden_layer_sizes:  [10, 10, 5] | learning_rate=0.5  Gamma=0.1  max_depth=7 |
| 2 | Penalty:“L1”  C= 0.1 | Kernel: “rbf”  Gamma=0.1  C=100 | n_estimators=60  max_features=2 | hidden_layer_sizes:  [10, 5] | learning_rate=0.05  Gamma=0.01  max_depth=9 |
| 3 | Penalty:“L2”  C= 0.1 | Kernel: “rbf”  Gamma=0.1  C=1 | n_estimators=70  max_features=3 | hidden_layer_sizes:  [5] | learning_rate=1  Gamma=10  max_depth=7 |
| 4 | Penalty:“L2”  C= 100 | Kernel: “rbf”  Gamma=0.1  C=0.1 | n_estimators=120  max_features=6 | hidden_layer_sizes:  [5] | learning_rate=0.5  Gamma=0.1  max_depth=9 |
| 5 | Penalty:“L2”  C= 100 | Kernel: “rbf”  Gamma=0.1  C=10 | n_estimators=30  max_features=6 | hidden_layer_sizes:  [5] | learning_rate=0.01  Gamma=1  max_depth=6 |
| 6 | Penalty:“L2”  C= 10 | Kernel:“rbf”  Gamma=0.1  C=10 | n_estimators=30  max_features=11 | hidden_layer_sizes:  [10, 5, 5] | learning_rate=0.05  Gamma=0.01  max_depth=10 |
| 7 | Penalty:“L2”  C= 0.1 | Kernel: “rbf”  Gamma=0.1  C=0.1 | n_estimators=110  max_features=3 | hidden_layer_sizes:  [10, 5, 5] | learning_rate=0.1  Gamma=0.01  max_depth=8 |
| 8 | Penalty:“L1”  C= 1 | Kernel:“rbf”  Gamma=0.1  C=0.1 | n_estimators=30  max_features=5 | hidden_layer_sizes:  [5, 5, 10] | learning_rate=0.05  Gamma=1  max_depth=7 |
| 9 | Penalty:“L1”  C= 0.1 | Kernel: “rbf”  Gamma=0.1  C=0.1 | n_estimators=80  max_features=4 | hidden_layer_sizes:  [10, 10, 10] | learning_rate=0.1  Gamma=0.01  max_depth=10 |
| 10 | Penalty:“L2”  C= 0.1 | Kernel: “rbf”  Gamma=1  C=0.1 | n_estimators=40  max_features=2 | hidden_layer_sizes:  [10, 10, 5] | learning_rate=0.05  Gamma=10  max_depth=5 |

For LR, the “penalty” was tuned from “none”, “L1” and “L2”; the “C” parameter is selected from 0.001, 0.1, 1, 10, 100. For SVM, the “kernel” parameter is searching from “linear” and “rbf”; the “gamma” parameters include 0.001, 0.1, 1, 10, 100; the “C” parameter is selected from 0.001, 0.1, 1, 10, 100. For RF, the “n_estimators” parameter is from 10 to 150 by 10; the “max_features” parameter is from 1 to 12. For ANN, the “hidden_layer_sizes” parameter is selected from “[5,5]”, “[10,10]”, “[5,10]”, “[10,5]”, “[10,10,10]”, “[5,5,5]”, “[5,10,10]”, “[10,5,10]”, “[5,10,5]” “[10,10,5]”, “[5,5,10]”, “[10,5,5]”. For XGboost, the “learning_rate” parameters include 0.01, 0.05, 0.1, 0.5, 1; the “gamma” parameter is selected from 0.01, 0.1, 1, 10; the “max_depth” parameter is from 1 to 10. All models’ hyper-parameters except the above are set to default values.
