# Supplementary material for: Correction: Interpretable classifiers for prediction of disability trajectories using a nationwide longitudinal database
Source: BMC Geriatr. 2023 Mar 30;23:190. doi: 10.1186/s12877-023-03843-z (PMC10064752; doi:10.1186/s12877-023-03843-z)
Supplement: Supplementary file 1 — Additional file 1. Revised content in main text. [file 12877_2023_3843_MOESM1_ESM.docx]

**Revised content in main text**

1. In Table 2 of the RESULTS section, some of the numbers for group comparisons are incorrect, we have revised it as follows.

- Age (Total sample) : 74.99±7.58 should be modified to 77.61±9.31
- Sex-Male (Total sample) : 1972 (46.4%) should be modified to 1927(46.4%)
- Education-Literate (Trajectory Class-Progressive): 227 (35.3%) should be modified to 227 (35.5%)
- Stroke-No (Total sample): 3946 (95.6%) should be modified to 3846 (95.6%)
- Household Income per Capita-High level (Trajectory Class-High-onset): 60 (3.4%) should be modified to 60 (21.7%)

1. In RESULTS section, the first paragraph of “*Baseline characteristics of study population*” part, some incorrect writings were revised.

**The original text**: Table 2 shows the results of baseline characteristics of the study samples with different trajectory classes. This study identified 3210, 642, and 297 participants with normal class, progressive class, and high-onset class, respectively. For the comparisons of baseline characteristics among trajectory classes, significant differences were found in all variables except for ethnicity, fruit intake, systolic pressure, length from wrist to shoulder, length from kneel to floor, PWB score, household income per capita, and sufficient financial support. Comparisons between the analytical sample and drop-out sample are presented in Supplementary Table 3. The samples in the current analysis were younger, more likely to live with family members and suffer from hypertension, had more females and rural residents, had lower frequency of fruit intake, had higher frequency of leisure activity and heart rate, had better basic activity of daily living, worse chronic condition, and a low level of household income.

**The words in red font should be corrected as follows.**

- “and sufficient financial support” should be modified to “diabetes, and sufficient financial support”.
- “heart rate” should be modified to “lower heart rate”
- “basic” should be modified to “instrumental”
- “worse” should be modified to “less”

1. In RESULTS section, the first paragraph of “*Performance evaluation of three-class prediction models*” part, one small error should be corrected.

**The original text**: The selected variables with LASSO in five sets of imputation data were shown in Supplementary Table 4, and the optimal hyper-parameters for each of the imputation data sets were listed in Supplementary Table 5. The performance of prediction models was shown in Table 3. Generally, the performance of five ML algorithms was comparable in the full-variable and selected-variable data sets. Specifically, XGBoost and RF achieved relatively the best performance, with balanced accuracy around 0.77, weighted recall around 0.84, weighted specificity and weighted F1 score nearly 0.85.

**The words in red font should be corrected as follows.**

- “specificity” should be modified to “precision”

1. In RESULTS section, the first paragraph of “Interpretability analysis of prediction models” part, one samll error should be corrected.

**The original text**: The global interpretability method in SHAP was used to interpret the decisions for RF and XGBoost. For three-class task, the top 20 predictors for overall prediction and for prediction of each specific trajectory were shown in Fig. 4A-D (RF) and Fig. 4E-H (XGBoost). The top five most important predictors of RF were IADL, age, leisure activity, MMSE, and BADL. In XGBoost, IADL, age, BADL, leisure activity, and systolic pressure were the top five predictors. For two-class prediction, the top 20 predictors for overall prediction and for abnormal trajectory prediction were shown in Fig. 5 A-B (RF) and Fig. 5 C-D (XGBoost).

**The words in red font should be corrected as follows.**

- “abnormal” should be modified to “specific”.

1. In RESULTS section, One error should be corrected in the figure legend of “*Fig. 5 | The relative feature importance (top 20) of RF (A-B) and XGBoost (C-D) in two-class prediction.*”.

**The original figure legend**: A: overall importance of RF; B: SHAP summary plot of RF model when the expected outcome is abnormal; C: overall importance of XGBoost; D: SHAP summary plot of XGBoost model when the expected outcome is abnormal.

**The words in red font should be corrected as follows.**

- “abnormal” should be modified to “normal”.
